# Acid Adaptation Promotes TRPC1 Plasma Membrane Localization Leading to Pancreatic Ductal Adenocarcinoma Cell Proliferation and Migration through Ca^2+^ Entry and Interaction with PI3K/CaM

**DOI:** 10.3390/cancers14194946

**Published:** 2022-10-09

**Authors:** Julie Schnipper, Sana Kouba, Frédéric Hague, Alban Girault, Marie-Sophie Telliez, Stéphanie Guénin, Ahmed Ahidouch, Stine Falsig Pedersen, Halima Ouadid-Ahidouch

**Affiliations:** 1 Laboratory of Cellular and Molecular Physiology, UR UPJV 4667, University of Picardie Jules Verne, 80000 Amiens, France; 2 Regional Ressources Center for Molecular Biology (CRRBM), University of Picardie Jules Verne, 80000 Amiens, France; 3 Biology Department, Sciences Faculty, University Ibn Zohr, Agadir 80000, Morocco; 4 Section for Cell Biology and Physiology, Department of Biology, University of Copenhagen, 2100 Copenhagen Ø, Denmark

**Keywords:** pancreatic ductal adenocarcinoma, tumor microenvironment, acid adaptation, Ca^2+^ signaling, TRPC1, cell migration, cell proliferation, spheroid growth, cell cycle progression, PI3K, calmodulin

## Abstract

**Simple Summary:**

Pancreatic ductal adenocarcinoma (PDAC) is one of the deadliest cancers globally, with a 5-year overall survival of less than 10%. The development and progression of PDAC are linked to its fluctuating acidic tumor microenvironment. Ion channels act as important sensors of this acidic tumor microenvironment. They transduce extracellular signals and regulate signaling pathways involved in all hallmarks of cancer. In this study, we evaluated the interplay between a pH-sensitive ion channel, the calcium (Ca^2+^) channel transient receptor potential C1 (TRPC1), and three different stages of the tumor microenvironment, normal pH, acid adaptation, and acid recovery, and its impact on PDAC cell migration, proliferation, and cell cycle progression. In acid adaptation and recovery conditions, TRPC1 localizes to the plasma membrane, where it interacts with PI3K and calmodulin, and permits Ca^2+^ entry, which results in downstream signaling, leading to proliferation and migration. Thus, TRPC1 exerts a more aggressive role after adaptation to the acidic tumor microenvironment.

**Abstract:**

Pancreatic ductal adenocarcinoma (PDAC) remains one of the most lethal malignancies, with a low overall survival rate of less than 10% and limited therapeutic options. Fluctuations in tumor microenvironment pH are a hallmark of PDAC development and progression. Many ion channels are bona fide cellular sensors of changes in pH. Yet, the interplay between the acidic tumor microenvironment and ion channel regulation in PDAC is poorly understood. In this study, we show that acid adaption increases PANC-1 cell migration but attenuates proliferation and spheroid growth, which are restored upon recovery. Moreover, acid adaptation and recovery conditions favor the plasma membrane localization of the pH-sensitive calcium (Ca^2+^) channel transient receptor potential C1 (TRPC1), TRPC1-mediated Ca^2+^ influx, channel interaction with the PI3K p85α subunit and calmodulin (CaM), and AKT and ERK1/2 activation. Knockdown (KD) of TRPC1 suppresses cell migration, proliferation, and spheroid growth, notably in acid-recovered cells. KD of TRPC1 causes the accumulation of cells in G0/G1 and G2/M phases, along with reduced expression of CDK6, −2, and −1, and cyclin A, and increased expression of p21^CIP1^. TRPC1 silencing decreases the basal Ca^2+^ influx in acid-adapted and -recovered cells, but not in normal pH conditions, and Ca^2+^ chelation reduces cell migration and proliferation solely in acid adaptation and recovery conditions. In conclusion, acid adaptation and recovery reinforce the involvement of TRPC1 in migration, proliferation, and cell cycle progression by permitting Ca^2+^ entry and forming a complex with the PI3K p85α subunit and CaM.

## 1. Introduction

Pancreatic ductal adenocarcinoma (PDAC) accounts for up to 90% of pancreatic cancer incidences, and is the seventh leading cause of cancer-related deaths worldwide [1,2]. The poor prognosis of PDAC patients is mainly caused by late diagnosis, at a stage where the disease is often advanced, metastatic, and non-resectable [3,4]. The cytotoxic therapies available at present are modestly effective, and there is a compelling need to explore underlying PDAC development and progression mechanisms to develop better treatment options [3]. 

The development and progression of PDAC is linked to the physiology and microenvironment of the exocrine pancreas. Pancreatic epithelial and stromal cells are exposed to cyclic changes in the extracellular pH (pH_o_) due to the production of alkaline pancreatic juices rich in HCO_3_^−^. The apical exposure to this alkaline pH_o_ is coupled to the parallel acidification of the basolateral membrane, leading to an acidic pancreatic interstitium [5,6]. Interestingly, extracellular acidification is one of the key characteristics of the tumor microenvironment, where it is caused by low perfusion in combination with high extrusion of H^+^ from fermentative glycolysis and acid in the form of CO_2_ from oxidative phosphorylation [7,8,9]. While the interstitial space of solid tumors is generally acidic, and pH_o_ values as low as 5.6 have been measured, most values are in the range of 6.4–7. Where regions of the tumor with poor vascularization will typically be acidic, well-vascularized areas will exhibit a pH_o_ closer to neutral [8,10,11,12]. The intracellular pH (pH_i_) is more alkaline than the pH_o_ in tumors, yet cancer cells in an acidic microenvironment still exhibit an acidic pH_i_ [13,14] compared to healthy cells in the normal pancreas.

Long-term acidosis acts as an evolutionary selection pressure. It causes adaptive responses that can establish cancer cell populations with more malignant phenotypes in the form of invasive and metastatic potential [8,15,16,17]. In addition, acidosis limits proliferation by keeping the cancer cells in a dormant state [18]. This adaptation can be of particular impact once the cancer cells encounter a more neutral microenvironment, as an increase in pH_i_ can further accelerate proliferation [8,9,19,20]. Changes in the pH can produce a multitude of cellular physiological effects, as numerous proteins are sensitive to pH in the range encountered in tumors [19,20,21]. Transmembrane proteins, such as ion channels, can function as pH sensors and transduce extracellular signals as changes in pH_o_ [19,22]. They can potentially regulate signaling pathways related to all hallmarks of cancer by being affected by both pH_o_ and pH_i_ [19,20,21]. In recent years, the role of calcium (Ca^2+^) channels as drivers of cancer mechanisms has been extensively investigated [23,24,25]. The transient receptor potential canonical (TRPC) channel subfamily represents a group of Ca^2+^-permeable non-selective cation channels [26]. Different physiochemical stimuli can activate their gating mechanisms and affect their expression, thus being categorized as cellular sensors [27]. The TRPC1 isoform is involved in various physiological and pathological processes [27,28] through different stimuli. For instance, the expression of TRPC1 increases and modulates proliferation and migration via hypoxia-induced signaling in breast and follicular thyroid cancer cells [29,30]. TRPC1 expression increases upon 24 h of pressure in pancreatic stellate cells (PSCs) [31,32]. The ambient pressure activates TRPC1 to form a complex with α smooth muscle actin (αSMA) and phosphorylated SMAD2. This physical interaction between TRPC1/αSMA/SMAD2 is essential for activating two major pathways underlying PSC activation, namely ERK1/2 and SMAD2 pathways, resulting in IL-6 secretion and PSC proliferation [32]. Interestingly, the TRPC1 plasma membrane expression decreases upon PI3K inhibition in glioblastoma cells, which is associated with reduced chemotaxis and cell migration [33]. Although the response of TRPC1 to acidification has, to our knowledge, not been reported, other TRPC channels have been shown to be affected by acidification [34]. For instance, TRPC4 and TRPC5 are activated by acidic pH in HEK293 cell models. TRPC4 is activated by acidic pH_i_ (6.75–6.25), in combination with increased cytosolic Ca^2+^ levels and G-protein-coupled receptor activation [35], while TRPC5 is activated both through G-protein activation and directly through protonation (pH 6.5) [36].

We have recently shown that TRPC1 is overexpressed in PDAC tissue and cell lines but does not regulate either basal Ca^2+^ entry or store-operated Ca^2+^ entry (SOCE). However, TRPC1 regulates PANC-1 cell proliferation by interacting with phosphoinositol-3-kinase (PI3K) and calmodulin (CaM) and regulating AKT signaling in normal pH conditions [37]. In the present study, we demonstrate the role of the fluctuating acidic tumor microenvironment and TRPC1 in PDAC cell migration, proliferation, and cell cycle progression. We showed that in acid-adapted PDAC cells, total TRPC1 expression decreased, but plasma membrane localization increased. In acid-recovered cells, TRPC1 expression increased, high plasma membrane localization of the channel was maintained, and Ca^2+^ influx increased. As we have demonstrated before, TRPC1 formed a complex with PI3K/CaM, activating the downstream serine/threonine protein kinase AKT, but in this study, TRPC1 also activated the extracellular signal-regulated kinase 1 and 2 (ERK1/2) that synergically control cell migration, proliferation, and cell cycle progression, in acid adaptation and recovery conditions.

## 2. Materials and Methods

### 2.1. Cell Culture and Experimental pH Setup

The normal duct-like cell line human pancreatic nestin-expressing cells (HPNE), immortalized with hTERT, was purchased from the American Type Culture Collection (ATCC, Molsheim, France). HPNE were grown in 75% DMEM without glucose (Sigma-Aldrich, Saint-Quentin-Fallavier, France), 25% Medium M3 Base (Incell Corp. Cat#M300F-500), and 10% fetal bovine serum (Cat#P30-3031, PAN Biotech), 5.5 mM glucose, and 750 ng/mL Puromycin. The human PDAC cell line PANC-1 cells were kindly provided by Prof. Anna Trauzold (Institute of Experimental Cancer Research, Kiel University, Kiel, Germany). PANC-1 cells were cultured in RPMI-1640 medium already containing glucose (Sigma-Aldrich, Saint-Quentin-Fallavier, France) supplemented with 10% fetal bovine serum (Cat#P30-3031, PAN Biotech), 1 mM sodium pyruvate (Gibco, Waltham, MA, USA), and 1% Glutamax (Gibco). Cells were grown at 37 °C, 95% humidity, 5% CO_2_, and passaged with trypsin-EDTA 0.25% (Sigma-Aldrich, Saint-Quentin-Fallavier, France) when cells reached a confluency of 70–80%. Cell cultures were not used for more than 20 passages. The medium pH was adapted to pH 6.5 by adjusting the HCO_3_^−^ concentration by adding the appropriate amount of NaHCO_3_ and ensuring equal osmolarity by changing [NaCl] as suggested by Michl et al., [38], and as performed previously by Flinck et al., Yao et al., and Hagelund and Trauzold [39,40,41]. All cell lines were regularly tested for mycoplasma. 

PANC-1 cells were thawed and grown under normal pH conditions (pH 7.4), then transferred to medium with pH 6.5, and maintained in this medium for a period of 30 days. These cells are referred to as acid-adapted cells (pH 6.5). The acid-adapted PANC-1 cells were then reseeded in a pH 7.4 medium for 14 days and are referred to as acid-recovered cells (pH 7.4R).

### 2.2. Live Imaging of Intracellular pH

As previously described, the pH_i_ measurements of non-transfected PANC-1 cells were performed [41,42]. In detail, 8 × 10^4^ cells were seeded in WillCo glass-bottom dishes WillCo Wells, Amsterdam, The Netherlands. After 48 h of seeding, cells were incubated in a growth medium containing 4 µM 2,7-bis-(2-carboxyethyl)-5-(and-6)-carboxyfluorescein acetoxymethyl ester (BCECF-AM, Invitrogen) for 30 min in the dark at 37 °C, 95% humidity, 5% CO_2_. Cells were washed twice in HCO_3_^−^ containing Ringer solution; 115 mM (for pH 7.4) or 135 mM (for pH 6.5) NaCl, 24 mM (for pH 7.4) or 3 mM (for pH 6.5) NaHCO_3_, 5 mM KCl, 1 mM MgSO_4_, 1 mM Na_2_HPO_4_, 1 mM CaCl_2_, 3.3 mM 3-(-N-morpholino) propanesulfonic acid (MOPS), 3.3 mM 2 [Tris(hydroxymethyl)-methylamino]-ethanesulfonic acid (TES), 5 mM 4-(2-hydroxyethyl)-1-piperazineethanesulfonic acid (HEPES), adjusted with NaOH or HCl to pH 7.4 or 6.5 at 37 °C. Then, the cells in the glass-bottom dish containing Ringer solution adjusted to the respective pH were placed in a temperature-controlled compartment (37 °C) equipped with gas. The steady-state pH_i_ measurements were carried out using a Nikon Eclipse Ti microscope equipped with a Xenon lamp for fluorescence excitation, a 40× oil 1.4 NA objective, and EasyRatioPro imaging software (PTI), for 10 min. Emission was measured at 520 nm after excitation at 440 nm and 485 nm. Calibration was carried out using the high K^+^/nigericin technique [43], employing KCl solutions (156 mM KCl, 1 mM MgSO_4_, 1 mM CaCl_2_, 1 mM K_2_HPO_4_, 3.3 mM MOPS, 3.3 mM TES, 5 mM HEPES) of pH 6.6, 7.0, 7.4, and 7.8, and 10 μM Nigericin (Sigma-Aldrich), which generated a four-point linear calibration curve to calibrate pH_i_ values. Fluorescence measured from the two excitation channels (440 nm and 485 nm) was corrected for their respective background fluorescence during each experiment. The background fluorescence was assessed by measuring in a cell-free area during the experiment. The ratio 485 nm/440 nm was then calculated, and the calibration data were fitted to a linear function in the applied pH range. The experimental data were inserted and converted to corrected steady-state pH values.

### 2.3. Transient Transfections

Cells were transfected with small interfering RNA (siRNA) by electroporation using nucleofection (Amaxa Biosystems, Lonza, Aubergenville, France) as previously described [37]. Briefly, PANC-1 cells (1 × 10^6^) grown under either normal pH, acid adaptation, or acid recovery conditions, were transiently nucleofected according to the manufacturer’s protocol. To achieve this, 4 μg of scrambled siRNA (siCTRL, negative duplex control, Eurogentec) or siRNA directed against TRPC1 (siTRPC1, ON-TARGET plus SMART pool siRNA, Dharmacon Research, Chicago, IL, USA) were used. Experiments were performed 72 h after siRNA transfection unless otherwise indicated. 

### 2.4. 3D Spheroid Growth and CellTiter-Glo Assay

Non-transfected or transfected PANC-1 cells grown under either normal or acid adaptation conditions were formed into spheroids in their respective medium or in normal pH conditions following acid adaptation to simulate the 2D model of acid recovery conditions. In total, 2000 PANC-1 cells were seeded in round-bottomed, ultralow attachment 96-well plates (Corning, NY, USA) in 200 μL growth medium, supplemented with 2% GelTrex LDEV-Free reduced growth factor basement membrane matrix (Thermo Fisher Scientific, Waltham, MA, USA). After seeding, cells were spun down at 750 RCF for 20 min and were grown for 9 days at 37 °C with 95% humidity and 5% CO_2_, and 100 μL of the respective pH medium was exchanged every second day. Light microscopic images of the spheroids at 10× magnification were acquired on days 2, 4, 7, and 9. On day 9, PANC-1 spheroids, either non-transfected or transfected cells (in replicates of three) were transferred to a black 96-well plate with 100 µL of the respective pH medium and 100 µL CellTiter-Glo^®^ 3D Reagent (Promega, Madison, WI, USA). This plate was wrapped in aluminum foil, shaken for 5 min, and then incubated without shaking for 25 min at room temperature. The luminescence signal was recorded using a FLUOStar Optima microplate reader (BMG Labtech, Ortenberg, Germany).

### 2.5. Western Blot Analysis

Proteins were extracted, determined, and separated by the SDS-page technique as previously described [44]. The primary antibodies used were: anti-TRPC1 (1:1000, Abcam, Waltham, MA, USA), anti-GAPDH (1:2000, Cell Signaling Tech., Danvers, MA, USA), anti-CDK6 (1:500, Cell Signaling Tech., Danvers, MA, USA), anti-Cyclin A (1:500, Santacruz Biotechnology, Dallas, TX, USA), anti-CDK2 (1:500, Cell Signaling Tech., Danvers, MA, USA), anti-CDK1 (1:500, Cell Signaling Tech., Danvers, MA, USA), anti-p21^CIP1^ (1:500, Cell Signaling Tech., Danvers, MA, USA), anti-PI3K p85α (1:500, Bioworld Technology, tebu-bio, France), anti-calmodulin (1:500, Santacruz Biotechnology, Dallas, TX, USA), anti-pAKT (Ser473) (1:500, Cell Signaling Tech., Danvers, MA, USA), anti-AKT (1:500, Cell Signaling Tech., Danvers, MA, USA), anti-pERK1/2 (Thr202/Tyr204) (1:500, Cell Signaling Tech., Danvers, MA, USA), and anti-ERK1/2 (1:500, Cell Signaling Tech., Danvers, MA, USA). Secondary antibodies (1:4000, Cell Signaling Tech., Danvers, MA, USA) were coupled to horseradish peroxidase, and proteins were detected using enhanced chemiluminescence (Ozyme, Saint-Cyr-l’Ecole, France). Quantification was performed with the ImageJ software 1.53a (National Institute of Health, Bethesda, MD, USA) analysis tool. All experiments were normalized to the level of GAPDH.

### 2.6. Cell Surface Biotinylation Assay

To determine the membrane fraction of TRPC1, 1 × 10^6^ HPNE cells or 8 × 10^5^ PANC-1 cells grown in normal pH, acid adaptation, or acid recovery conditions were seeded in 60 mm Petri dishes for 48 h and collected as previously described [45]. Briefly, cells were washed three times with cold PBS, then incubated with 3 mg of sulfo-NHS-SS-biotin (Thermo Fisher Scientific) and slightly shaken for 1 h at 4 °C. The reaction was interrupted by the addition of cold PBS containing 10 mM glycine. Cells were scrapped with RIPA buffer, and ~10% of the total lysate was saved as the total lysate fraction. The remaining lysate portion (corresponding to the membrane fraction) was incubated with high-capacity streptavidin agarose beads (Thermo Fisher Scientific, Waltham, MA, USA) with gentle rotation overnight at 4 °C. After incubation, beads were washed four times with RIPA buffer. Proteins were eluted from the beads with 50 µL of Laemmli buffer 2X and heated at 60 °C for 30 min. Both total lysate samples and membrane fraction samples were used for Western blot analysis, as described above.

### 2.7. Co-Immunoprecipitation

The 6 × 10^5^ non-transfected or 1 × 10^6^ transfected PANC-1 cells grown in the three different pH conditions were seeded in 10 cm Petri dishes and collected after 72 h. As previously described [37], 500 µg of proteins were used for co-immunoprecipitation with SureBeads™ Protein A Magnetic Beads (Bio-Rad, France). Beads were washed thoroughly, according to the manufacturer’s protocol. Then, 1 µg of either TRPC1 antibody (Abcam, Waltham, MA, USA), PI3K p85α antibody (Bioworld Technology, tebu-bio, France), or a control HRP-linked anti-rabbit IgG antibody (Cell Signaling Tech., Danvers, MA, USA) were resuspended with the beads for 30 min. Protein lysates were subsequently washed and added to the beads, which were slowly rotated for 2 h at room temperature. After another sequential washing step of the beads, proteins were eluted according to the manufacturer’s protocol. After denaturation, proteins were subjected to Western blotting as described above. To detect the input, 50 µg of proteins from the corresponding co-immunoprecipitation samples were used.

### 2.8. Immunofluorescence Assay and Analysis

The 8 × 10^4^ non-transfected PANC-1 cells, grown under the three different pH conditions, were seeded on coverslips for 48 h. Immunofluorescent staining was performed as previously described [37]. Briefly, cells were washed in cold PBS and fixed for 20 min at room temperature in 4% paraformaldehyde (PFA, Sigma, Saint-Quentin-Fallavier, France). Cells were washed twice in PBS and permeabilized in 0.1% Triton^TM^X-100 (Sigma, Saint-Quentin-Fallavier, France) for 10 min. Next, cells were blocked in 5% bovine serum albumin (BSA, Sigma, Saint-Quentin-Fallavier, France) for 30 min, followed by the addition of primary antibodies overnight at 4 °C. The antibodies used were anti-TRPC1, (1:100, Santacruz Biotechnology, Dallas, TX, USA), with Na^+^/HCO_3_^−^ co-transporter (NBCn1) used as a membrane marker (1:400, Abcam, Waltham, MA, USA), and anti-PI3K p85α (1:100, Bioworld Technology, tebu-bio, France). Secondary antibodies (AlexaFluor^®^ 488/550 conjugated antibody 1:600) were applied for 1 h at room temperature, followed by treatment with 4′,6-diamidino-2-phenylindole (DAPI; 1%) for 5 min to stain nuclei. Coverslips were washed three times and mounted on slides using Prolong^®^ Gold antifade reagent. Images were collected on an Olympus Cell Vivo IX83 with a Yokogawa CSU-W1 confocal scanning unit. Z-stacks were deconvoluted in Olympus Cell Sens software using a constrained iterative algorithm. No or negligible labeling was seen in the absence of primary antibodies. Overlays and brightness/contrast/background adjustments were carried out using ImageJ software. Mander’s overlapping R coefficient was calculated using the ImageJ software plugin JACoP, which calculates the proportion of the green signal coincident with the magenta signal over the total intensity [46]. The threshold setting was the same for both images. 

### 2.9. Boyden Chamber Assay

Cell migration was evaluated using a Boyden chamber assay with 8 μm pore size cell culture inserts (Falcon^®^, Corning, Boulogne-Billancourt, France), as previously described [47]. Briefly, 4 × 10^4^ non-transfected or transfected PANC-1 cells, grown under the three different pH conditions, were seeded in the upper compartment of the chamber. Both the upper and lower compartments were filled with the respective culture medium containing 10% FBS. After 24 h of incubation at 37 °C, 95% humidity, and 5% CO_2_, inserts were washed in PBS and fixed in methanol for 15 min at room temperature, followed by staining with hematoxylin for 5 min. Inserts were washed in Milli-Q water and cleaned with a cotton swab; 20 adjacent fields were counted per insert at ×400 magnification. The number of migrating cells was normalized to their respective control (normal pH conditions (pH 7.4) for non-transfected PANC-1 cells and siCTRL in normal pH conditions (pH 7.4) for transfected PANC-1 cells). To ensure that there was no difference in viability between the conditions after 24 h, the trypan blue assay (as described below) was performed.

To investigate the effect of extracellular Ca^2+^ concentrations on migration, we used the same Boyden chamber procedure as described above, but after 8 h of seeding, medium in the upper and lower chamber was changed to the respective medium, either with standard conditions containing 1 mM Ca^2+^ (referred to as conditions with Ca^2+^) or containing ethylene glycol tetraacetic acid (EGTA), to chelate Ca^2+^ and to end with a final concentration of 30 µM (referred to conditions without Ca^2+^). Thus, cells were transfected for 72 h, where Ca^2+^ where chelated for 24 h in total during the migration process.

### 2.10. Trypan Blue Assay

Non-transfected (4 × 10^4^) or transfected (8 × 10^4^) PANC-1 cells were seeded in 35 mm Petri dishes. Subsequently, 24, 48, 72, or 96 h after seeding, cells were washed in PBS, trypsinized, and diluted (1:5) in trypan blue solution (Sigma, Saint-Quentin-Fallavier, France). All conditions were counted six times using the standard Malassez cell method. Proliferation was calculated as the total number of viable cells (alive/white cells) normalized to the control. As previously described, we tested the effect of extracellular Ca^2+^ concentrations on proliferation [37]. Here, the same counting procedure as described above was carried out, but after 24 h of seeding, the medium was changed to medium with standard conditions containing 1 mM Ca^2+^ (referred to as conditions with Ca^2+^) or containing EGTA to chelate Ca^2+^ and to end with a final concentration of 30 µM (referred to conditions without Ca^2+^). Cells were transfected for 72 h, and Ca^2+^ was chelated for 48 h.

### 2.11. Flow Cytometry

Flow cytometry was carried out as described previously [37]. Briefly, duplicates of 2 × 10^5^ non-transfected or transfected PANC-1 cells were seeded in 60 mm Petri dishes and collected after 72 h. Cells were washed in PBS, trypsinized, and collected in PBS + EDTA (5 mM), followed by fixation with cold absolute ethanol (≥99.8%, Sigma, Saint-Quentin-Fallavier, France). Cells were kept at 4 °C for at least 6 h post-fixation. To prepare for analysis, cells were pelleted, resuspended in PBS + EDTA (5 mM), treated with 20 µg/mL RNase A (Sigma-Aldrich, St. Quentin Fallavier, France) for 30 min at room temperature, and stained with 50 µg/mL of propidium iodide (Sigma-Aldrich, St. Quentin Fallavier, France). The cell cycle distribution of each sample was determined by flow cytometry analysis of nuclear DNA content using a flow cytometer (Accuri^®^, Dominique Deutscher, Brumath, France). The cell percentage in different phases was calculated using Cyflogic software and illustrated with FCS Express 7.

### 2.12. Proximity Ligation Assay

The 8 × 10^4^ non-transfected or transfected PANC-1 cells grown in one of the three pH media were seeded on coverslips 72 h before the proximity ligation assay (PLA) experiment. As described before [47], cells were washed twice with PBS, then fixed with PFA (4%) at room temperature for 20 min. Cells were washed twice in PBS and permeabilized with 0.1% TritonTM X-100 (Sigma, Saint-Quentin-Fallavier, France) for 10 min. The Duolink in situ PLA detection kit (Sigma-Aldrich, Saint-Quentin-Fallavier, France) was used to detect interactions between TRPC1 and the PI3K p85α subunit. Experiments were performed according to the manufacturer’s protocol. Red fluorescent oligonucleotides produced as the end product of the procedure were visualized using the Zeiss Observer Z1 microscope 60X oil objective (Carl Zeiss, Oberkochen, Germany). Images were analyzed using ImageJ, where puncta per cell were counted and normalized to the respective control; normal pH conditions (pH 7.4) for non-transfected cells, and siCTRL in their respective medium (pH 6.5 or 7.4R). A total of 20 pictures per condition were captured and analyzed, and are presented as relative number puncta/cell.

### 2.13. Manganese Quench Assay

To estimate divalent cation influx under basal conditions, we used the manganese (Mn^2+^) quenching technique as previously described [47]. Briefly, 25 × 10^3^ transfected PANC-1 cells, grown under one of the three pH conditions, were seeded on glass coverslips for 72 h. At the beginning of each experiment, cells were incubated with 3.33 μM Fura-2/AM (Sigma, Saint-Quentin-Fallavier, France) at 37 °C, 95% humidity, 5% CO_2_ for 45 min in the dark. Cells on the coverslip were washed twice with extracellular saline solution (145 mM NaCl, 5 mM KCl, 2 mM CaCl_2_, 1 mM MgCl_2_, 5 mM glucose, 10 mM HEPES, pH 7.4 or pH 6.5), and placed on the stage of a fluorescence microscope (Axiovert 200; Carl Zeiss, Oberkochen, Germany). Cells were excited at 360 nm using a monochromator (polychrome IV, TILL Photonics, Gräfelfing, Germany), and fluorescent emission was captured with a Cool SNAPHQ camera (Princeton Instruments, Lisses, France) after filtration through a long-pass filter (510 nm). Metafluor software (version 7.1.7.0, Molecular Devices, San Jose, CA, USA) was used for signal acquisition and data analysis. After 1.5 min, the saline solution (including 2 mM Ca^2+^) was replaced by 2 mM Mn^2+^ solution by perfusion. The Mn^2+^ quenching extracellular solution contains 145 mM NaCl, 5 mM KCl, 2 mM MnCl_2_, 1 mM MgCl_2_, 5 mM glucose, and 10 mM HEPES, and was adjusted with NaOH or HCl to pH 7.4 or 6.5. The Mn^2+^ influx, a corroborate of Ca^2+^ influx, was estimated from the quenching rate of fluorescence at 360 nm by calculating the slope.

### 2.14. Statistical Analysis

All data are shown as representative images or as mean measurements with standard error of means (SEM) error bars and represent at least three independent experiments. *N* refers to the number of independent experiments performed, or to the number of cells analyzed. Welch’s correction *t*-test or Tukey’s multiple comparison test was applied to test for statistically significant differences between two groups. *, **, and *** denotes *p* < 0.05, *p* < 0.01, and *p* < 0.001, respectively. All graphs and statistics were generated in GraphPad Prism 9.0 software (San Diego, CA, USA).

## 3. Results

### 3.1. Acid Adaptation Promotes Membrane Localization of TRPC1 in PANC-1 Cells

**Figure 1:**
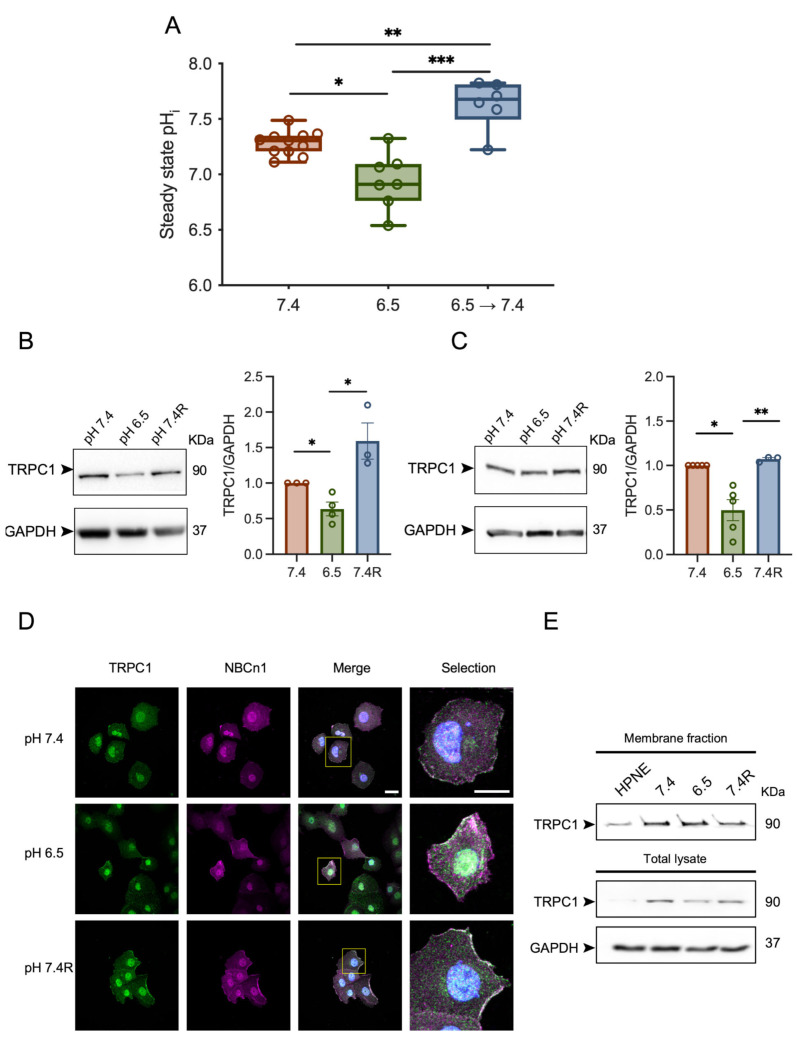
TRPC1 protein expression is affected by acid adaptation and recovery and favors the plasma membrane localization. (**A**) Steady state pH_i_ in PANC-1 cells grown in normal pH or acid adaptation conditions and measured in HCO_3_^−^ Ringer adjusted to pH 7.4 or 6.5 (X-axis). 6.5 → 7.4 indicate acid-adapted cells measured in pH_e_ 7.4. (*n* = 6–11). (**B**) Western blot analysis (left panel) and quantification (right panel) of TRPC1 expression in PANC-1 cells grown under normal pH (7.4), acid adaptation (6.5) or acid recovery conditions (7.4R) (*n* = 3–4). (**C**) Western blot analysis (left panel) and quantification (right panel) of TRPC1 expression in PANC-1 spheroids grown under normal pH (7.4), acid adaptation (6.5), or acid recovery conditions (7.4R) for 9 days (*n* = 3–5). Welch’s correction *t*-test was used to determine the significant difference between different conditions. *, ** and *** indicate *p* < 0.05 and 0.01 and 0.001 respectively. (**D**) Representative immunofluorescent analysis of TRPC1 and the membrane protein Na^+^/HCO_3_^−^ co-transporter (NBCn1, used as membrane marker) in PANC-1 cells grown under normal pH (7.4), acid adaptation (6.5) or acid recovery conditions (7.4R) (*n* = 3), scale bars = 20 µm. (**E**) Cell surface biotinylation followed by Western blotting indicating membrane fraction or total lysate of TRPC1. The uncropped blots are shown in File S1.

Before investigating the impact of the acidic tumor microenvironment on the growth and migration of PANC-1 cells, we studied the pH_i_ values in different pH_o_ conditions buffered with HCO_3_^−/^CO_2_. We observed that PANC-1 cells grown in normal pH conditions (pH 7.4) exhibited a pH_i_ of 7.3. This value decreased to 6.9 in acid-adapted cells (pH 6.5), and increased to 7.6 in acid-adapted cells measured at pH 7.4 (*n* = 6–11, *p* < 0.05, Figure 1A). 

Recently, we have shown an overexpression of TRPC1 in human PDAC tissue and the aggressive PANC-1 cell line [37]. Hence, we investigated the effect of the acidic tumor microenvironment on the expression and the localization of TRPC1 in PANC-1 cells. The protein levels of TRPC1 were significantly reduced by 37 ± 9% in acid-adapted PANC-1 cells compared to cells cultured in normal conditions (*n* = 3–4, *p* < 0.05, Figure 1B). Moreover, TRPC1 protein expression in acid-recovered PANC-1 cells (pH 7.4R) was significantly increased by 60 ± 16.9% compared to acid-adapted PANC-1 cells (*n* = 3–4, *p* < 0.05, Figure 1B). The protein levels of TRPC1 in spheroid PANC-1 cells grown in different pH conditions were comparable with those found in the 2D model (*n* = 3–5, *p* < 0.05, Figure 1C). Using surface biotinylation and confocal imaging, we found that TRPC1 was expressed in the plasma membrane of PANC-1 cells in all three pH conditions compared to a duct-like cell line HPNE. However, the membrane fraction was increased in the majority of acid-adapted cells (*n* = 3, Figure 1D,E). These results indicate that acid adaptation decreases the global expression of TRPC1, but favors its plasma membrane localization.

### 3.2. The Knockdown of TRPC1 Inhibits Cell Migration and the Growth of PANC-1 Cells and Spheroids under Acid Recovery Conditions

**Figure 2:**
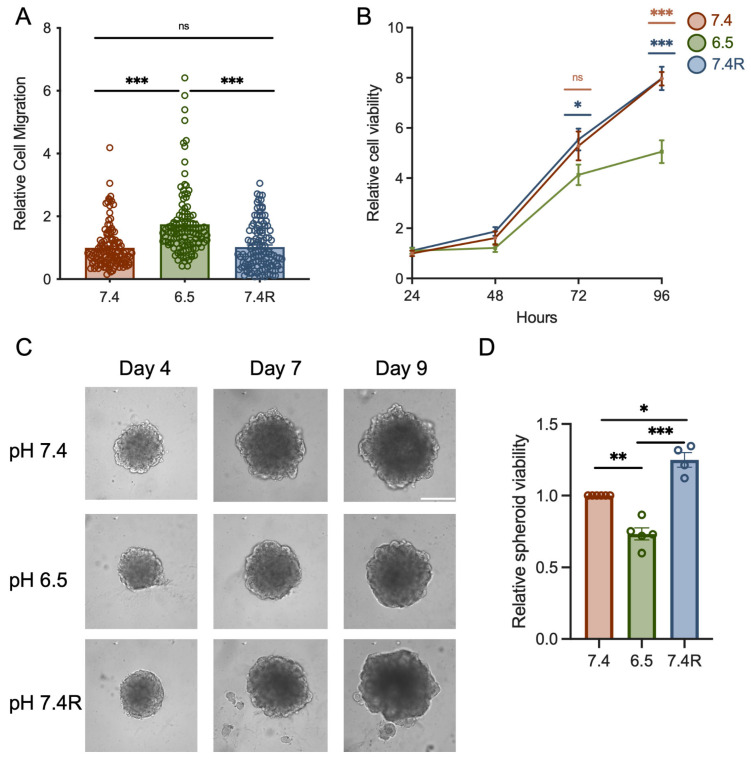
Acid adaptation promotes PANC-1 cell migration but attenuates proliferation, which is restored upon acid recovery. (**A**) Cell migration of PANC-1 cells grown under normal pH (7.4), acid-adapted (6.5), or acid recovery conditions (7.4R) analyzed by Boyden chamber assay (*n* = 3). (**B**) Trypan blue analysis of PANC-1 cells grown under normal pH (7.4), acid-adapted (6.5), or acid recovery conditions (7.4R) shows the relative number of viable cells after 24, 48, 72, and 96 h after seeding (*n* = 3). (**C**) Representative images of PANC-1 spheroids grown for 9 days and (**D**) viability quantification performed with CellTiter-Glo^®^ assay (*n* = 4–5), scale bar = 400 µm. Welch’s correction *t*-test was used to determine the significant difference between different conditions. ns indicates non-significance. *, **, and *** indicate *p* < 0.05, 0.01, and 0.001, respectively.

It is well known that the acidic tumor microenvironment can promote migration and slow down the proliferation of cancer cells [8,41,48,49]. First, we investigated the effect of pH on cell migration and viability. As expected, we found that acid-adapted PANC-1 cells migrated more than cells grown in normal pH and acid recovery conditions (75 ± 11.4 %, *n* = 3, *p* < 0.001, Figure 2A), and showed a significant decrease in viability (25 ± 10.7% and 36 ± 6.3% for 72 h and 96 h of cell culture, respectively) when compared to cells cultured in pH 7.4 (*n* = 3, *p* < 0.05, Figure 2B). Similar results were found in PANC-1 spheroids (*n* = 4, *p* < 0.01, Figure 2C,D). Indeed, acid-adapted PANC-1 spheroids grown for 9 days displayed lower viability by 25 ± 4% and by 43 ± 4.8% compared to normal pH and recovery conditions, respectively (*n* = 4–5, *p* < 0.01, Figure 2C,D). 

**Figure 3:**
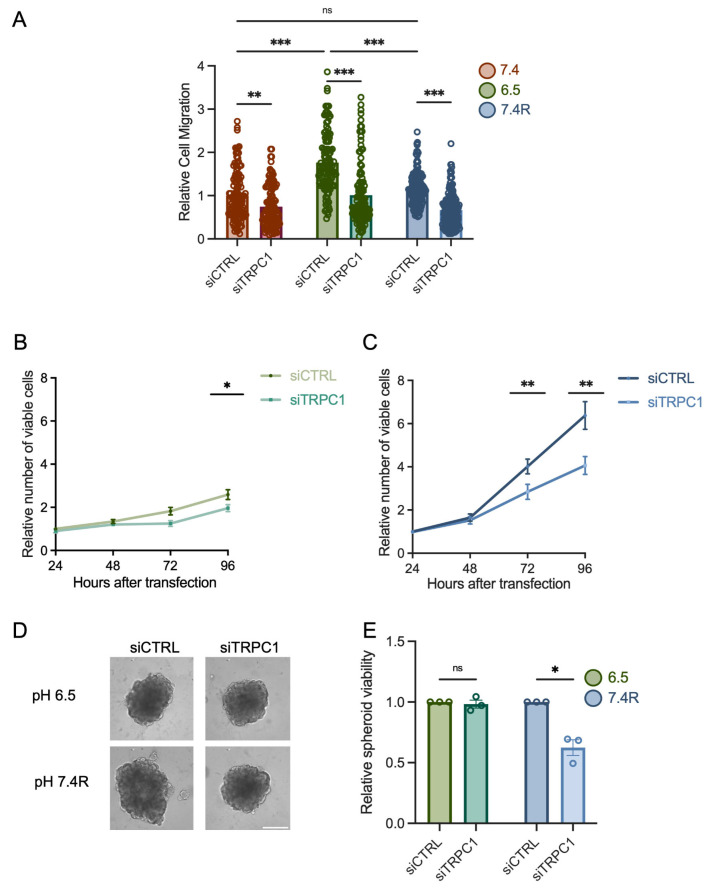
TRPC1 silencing inhibits cell migration, proliferation, and the growth of PANC-1 spheroids under acid recovery conditions. (**A**) Cell migration of siTRPC1 cells grown under normal pH (7.4), acid adaptation (6.5), or acid recovery conditions (7.4R) analyzed by Boyden chamber assay (*n* = 3–4). Tukey’s multiple comparison test was used to determine significant differences between conditions. (**B**) Trypan blue analysis of siTRPC1 PANC-1 cells grown under acid adaptation (6.5), or (**C**) acid recovery conditions (7.4R), showing the relative number of viable cells 24, 48, 72, and 96 h post-transfection (*n* = 3–5). (**D**) Representative images of siTRPC1 transfected PANC-1 spheroids grown for 9 days, and (**E**) viability quantification performed with CellTiter-Glo^®^ assay of siTRPC1 transfected spheroids grown under acid adaptation (6.5) or acid recovery (7.4R) (*n* = 3), scale bar = 400 µm. Welch’s correction *t*-test was used to determine the significant difference between siCTRL and siTRPC1 conditions. ns indicates non-significance. *, **, and *** indicate *p* < 0.05, 0.01, and 0.001, respectively.

To investigate the role of TRPC1, we validated our knockdown (KD) model. In normal pH, TRPC1 protein expression was reduced by 44 ± 10% 72 h post-transfection [37] and by 46 ± 3% and 59 ± 6.6% in acid adaptation and recovery conditions, respectively (Figure S1C). Similar results were found at the transcriptional level after 48, 72, and 96 h (Figure S1A,B). Silencing of TRPC1 reduced cell migration by 25 ± 6.8% in normal pH, 43 ± 5.1% in acid adaptation, and 49 ± 3.7% in acid recovery conditions (*n* = 3–4, *p* < 0.05 and 0.001, Figure 3A). We did not observe a significant effect of TRPC1 KD on viability after 24 h of seeding (Figure S2A). KD of TRPC1 in PANC-1 cells in normal pH conditions inhibited cell proliferation by 48 ± 17.4% and 38 ± 17.6% after 72 and 96 h, respectively [37], and spheroid growth by 22 ± 2.3% [37]. KD of TRPC1 decreased the viability of acid-adapted and -recovered cells by 31.5 ± 12.2% (*n* = 3–4, *p* < 0.05, Figure 3B) and 33 ± 12%, respectively 72 h post-transfection (n = 5, *p* < 0.01, Figure 3C). In addition, the annexin-5 analysis did not show any significant effect on apoptosis or necrosis (Figure S2E,F). To investigate whether the acid adaptation and recovery emphasize the involvement of TRPC1 in spheroid growth, we developed a siRNA-based KD of TRPC1 in spheroids. First, we confirmed the KD of TRPC1 after 9 days, where TRPC1 protein levels were decreased by 27 ± 16% and 41 ± 8% in acid adaptation and recovery conditions, respectively (Figure S1D). This KD of TRPC1 significantly decreased the viability of spheroids by 38 ± 6.5% in acid recovery conditions (*n* = 3, *p* < 0.05, Figure 3D,E). However, the silencing of TRPC1 did not affect the viability of acid-adapted spheroids (*n* = 3, Figure 3D,E). These results indicate that TRPC1 contributes to cell migration and proliferation of PANC-1 cells considerably in acid recovery conditions.

### 3.3. Knockdown of TRPC1 Accumulates Cells in the G0/G1 Phase and Decreases the Number of Cells in the G2/M Phase

**Figure 4:**
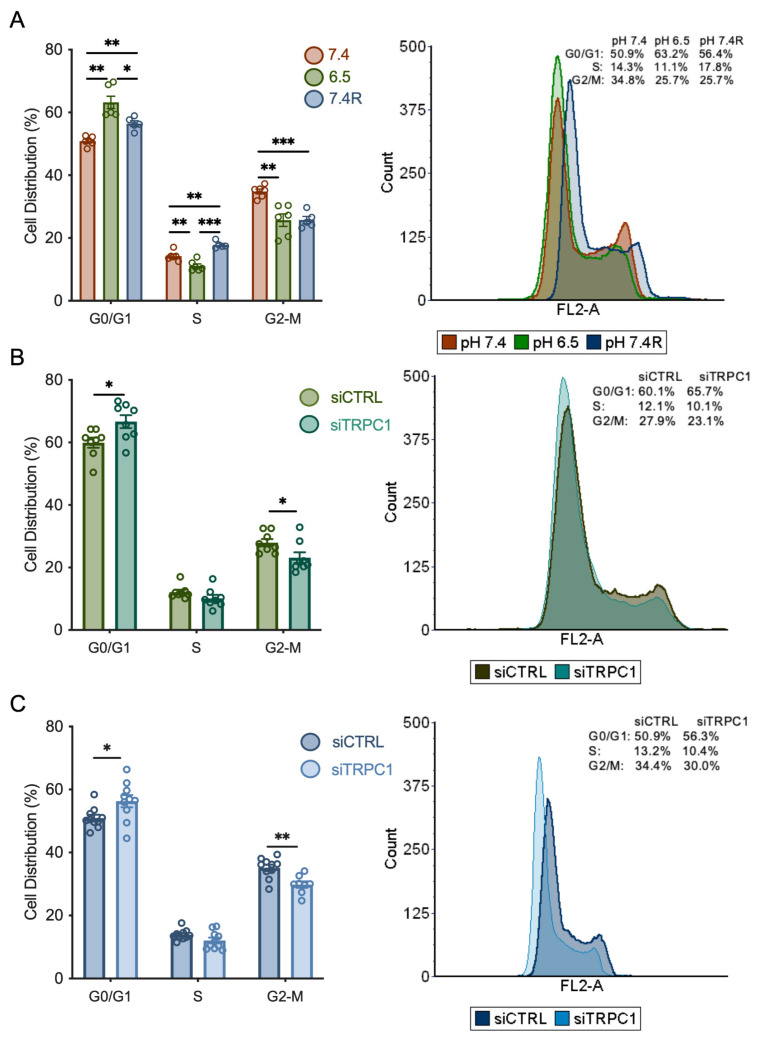
TRPC1 silencing accumulates cells in the G0/G1 phase and reduces the number of cells in the G2/M phase in both acid adaptation and recovery conditions. (**A**) Quantification of cell cycle analysis representing the percentage of cells in each cell cycle phase of non-transfected PANC-1 cells grown under normal pH (7.4), acid adaptation (6.5) or acid recovery conditions (7.4R) (left panel) and representative data from FACS acquisition (right panel). (**B**) Quantification of cell cycle analysis representing the percentage of cells in each cell cycle phase of siTRPC1 transfected PANC-1 cells grown under acid adaptation (6.5) or (**C**) acid recovery conditions (7.4R) (left panel), and representative data from FACS acquisition (right panel). Welch’s correction *t*-test was used to determine the significant difference between different conditions. *, **, and *** indicate *p* < 0.05, 0.01, and 0.001, respectively.

To investigate the mechanism by which pH and TRPC1 KD affect PANC-1 cell proliferation, we examined the cell cycle distribution by flow cytometry. First, we discovered that non-transfected cells grown in both acid adaptation and recovery conditions accumulated in the G0/G1 phase compared to cells cultured under normal pH conditions by 13.8 ± 4% and 10.7 ± 2.2%, respectively (*n* = 3, *p* < 0.01, Figure 4A). Furthermore, the number of cells in the S phase decreased significantly by 21 ± 6.4% in acid adaptation conditions. It increased significantly by 17 ± 5% in acid recovery conditions when compared to cells in normal pH conditions (*n* = 3, *p* < 0.01, Figure 4A). In the G2-M phase, the number of cells were decreased by 26 ± 5.8% and 26 ± 3.8% in acid adaptation and recovery conditions, respectively, compared to normal pH conditions (*n* = 3, *p* < 0.01 and 0.001, Figure 4A). These results indicate that acid adaptation of PANC-1 cells arrests them in the G0/G1 phase. In contrast, when they recover from this acid adaptation, they accumulate in the S phase and proliferate to a greater extent than cells grown in control conditions.

In addition to the effect of pH, we investigated the role of TRPC1 in the cell cycle distribution in acid-adapted and -recovered cells. We have previously shown that silencing of TRPC1 accumulated cells grown in normal pH conditions in the G0/G1 phase and decreased the number of cells in the S phase [37]. Here, we show, in the acid adaptation conditions, that TRPC1 silencing resulted in a slight accumulation (10 ± 4.3%) of cells in the G0/G1 phase and a decrease in the number of cells in the G2/M phase by 17 ± 7.4% (*n* = 4, *p* < 0.05, Figure 4B). This profile was maintained upon TRPC1 KD in acid recovery conditions; with an increase in the number of cells by 9.4 ± 4% in the G0/G1 phase and a decrease of 15.1 ± 4% in the G2/M phase (*n* = 4, *p* < 0.05 and 0.01, Figure 4C). These results suggest that TRPC1 is involved in G0/G1 progression, regardless of the pH condition. Furthermore, the role of TRPC1 in cell cycle progression shifts from the S phase in normal pH conditions to the G2/M phase in acidic and recovery conditions. 

### 3.4. TRPC1 Strongly Modulates the Expression of CDKs and Cyclin A in Acid Adaptation and Recovery Conditions 

**Figure 5:**
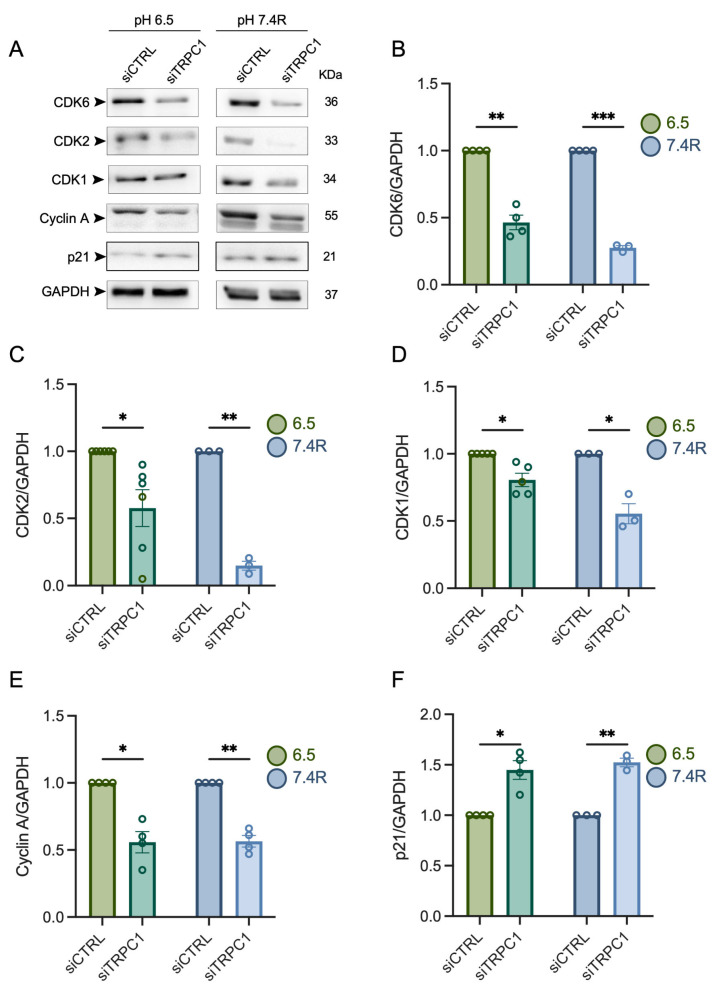
TRPC1 silencing decreases the expression of CDK6, -1, -2, and cyclin A and increases the expression of p21^CIP1^ to a greater extent in acid recovery conditions. (**A**) Western blot analysis of relevant cyclin-dependent kinase complexes and cyclins and their inhibitor p21^CIP1^ from cells grown under acid adaptation (6.5) or acid recovery conditions (7.4R). (**B**) Quantification of Western blot analysis representing the expression of CDK6, (**C**) CDK2, (**D**) CDK1, (**E**) cyclin A, and (**F**) p21^CIP1^ in siTRPC1 lysates compared to siCTRL lysates from PANC-1 cells grown under acid adaptation (6.5) or acid recovery (7.4R) conditions (*n* = 3 − 6). Welch’s correction *t*-test was used to determine the significant difference between siCTRL and siTRPC1. *, **, and *** indicate *p* < 0.05, 0.01, and 0.001, respectively. The uncropped blots are shown in File S1.

In normal pH conditions, the KD of TRPC1 reduced the expression of CDK6, CDK2, and cyclin A and increased p21^CIP1^ expression [37]. We investigated the effect of siTRPC1 on the expression of these proteins in both acid-adapted and -recovered cells. Our immunoblot analysis showed that the expression of CDK6 and CDK2 was more affected in acid-adapted and -recovered cells depleted of TRPC1. CDK6 protein expression was decreased by 53 ± 5.3% and 73 ± 1.5% and CDK2 expression was decreased by 43 ± 1.3% and 70 ± 1.5% in acid-adapted (*n* = 3–4, *p* < 0.01 and 0.001, Figure 5A,B) and -recovered cells (*n* = 3–6, *p* < 0.05 and 0.01, Figure 5A,C), respectively. A similar profile was observed for cyclin A and p21^CIP1^ expression. The expression of cyclin A was reduced by 45 ± 7.8% and 44 ± 4.2%, and the expression of p21^CIP1^ was upregulated by 31% ± 6.2% and 34% ± 2.7%, in acid-adapted and -recovered cells, respectively (*n* = 4, *p* < 0.05 and 0.01, Figure 5A,E,F). The expression of CDK1, the regulator driving cells through the G2/M phase, was not affected by TRPC1 KD in normal pH conditions [37]. In this study, the silencing of TRPC1 decreased CDK1 expression by 20 ± 4.9% and 45 ±1.4% for cells grown in acid adaptation and recovery conditions, respectively (*n* = 3–5, *p* < 0.05, Figure 5A,D). Moreover, TRPC1 KD failed to affect the expression of cyclin B1, D1, D3, and E, along with CDK4 in both acid-adaptated and -recovered cells (Figure S3A). Collectively, these results suggest that the depletion of TRPC1 has a stronger effect on the expression of the cell cycle regulating proteins, CDK1, -2, and -6, cyclin A, and p21^CIP1^ in acid-recovered PANC-1 cells than in normal pH and acid-adapted cells.

### 3.5. TRPC1 Interacts Strongly with the PI3K p85α Subunit and CaM under Acid Adaptation and Recovery Conditions

**Figure 6:**
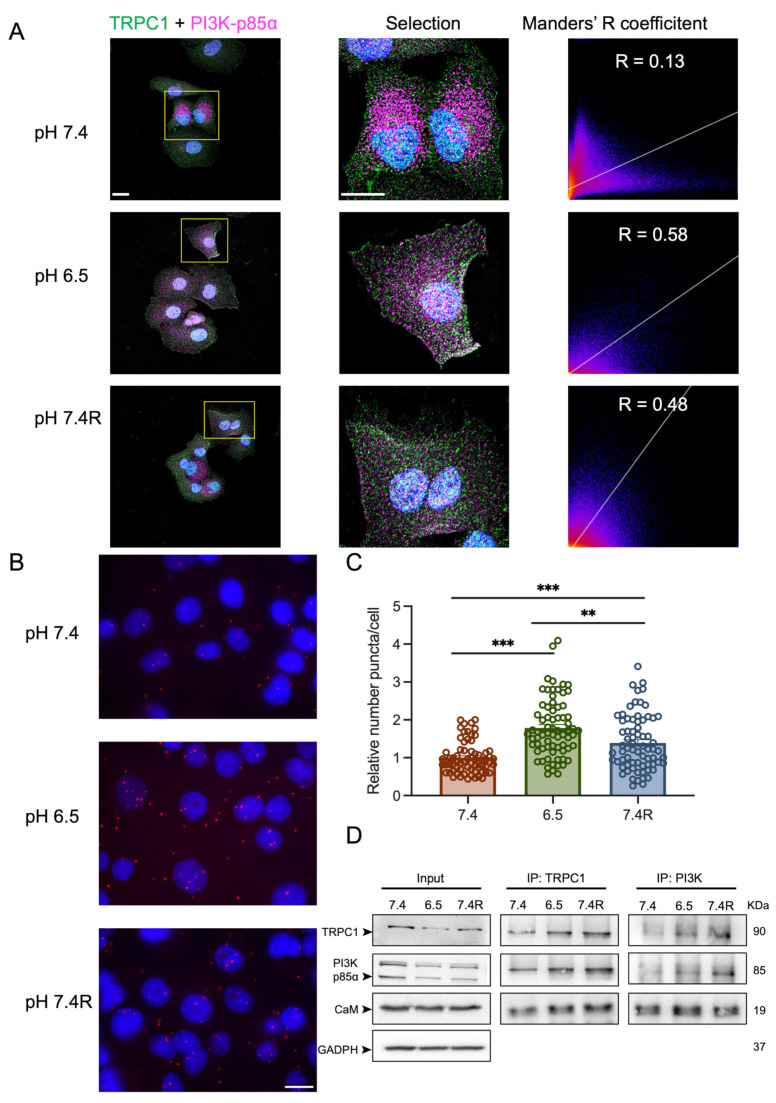
TRPC1 strongly interacts with the PI3K p85α subunit and CaM in acid adaptation and recovery conditions. (**A**) Representative immunofluorescent analysis of TRPC1 and the PI3K p85α subunit in PANC-1 cells grown under normal pH (7.4), acid adaptation (6.5) or acid recovery conditions (7.4R), scale bars = 20 µm. Mander’s R coefficient was used to quantify the co-localization between the two fluorophores (*n* = 3). (**B**) Representative images of proximity ligation assay (PLA) in PANC-1 cells grown under normal pH (7.4), acid adaptation (6.5), or acid recovery conditions (7.4R), scale bar = 10 µm. (**C**) Quantification of PLA where conditions are normalized to normal pH conditions (7.4) (*n* = 3). Welch’s correction *t*-test was used to determine the significant difference between different conditions. ** and *** indicate *p* < 0.01 and 0.001, respectively. (**D**) Representative Western blot analysis of co-immunoprecipitation of TRPC1 and PI3K p85α subunit with CaM in non-transfected PANC-1 cells grown under normal pH (7.4), acid adaptation (6.5) or acid recovery conditions (7.4R), (*n* = 3–5). The control pull-down performed with rabbit IgG is presented in Figure S3B. The uncropped blots are shown in File S1.

**Figure 7:**
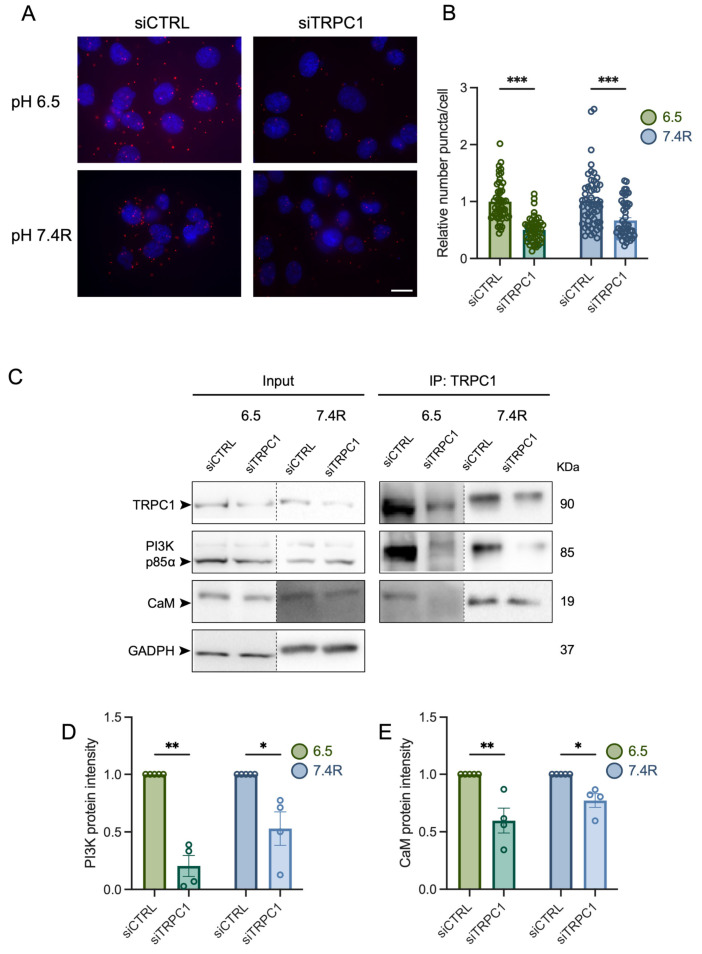
TRPC1 silencing inhibits the interaction between the PI3K p85a subunit and CaM. (**A**) Representative images of PLA in transfected PANC-1 cells grown under acid adaptation (6.5) or acid recovery conditions (7.4R). Scale bar = 10 µm. (**B**) Quantification of PLA in transfected PANC-1 cells grown under acid adaptation (6.5) or acid recovery conditions (7.4R), where siTRPC1 is compared to the relative number of siCTRL (*n* = 3). Welch’s correction *t*-test was used to determine the significant difference between different conditions. (**C**) Representative Western blot analysis of co-immunoprecipitation of TRPC1 with PI3K p85α and CaM in transfected PANC-1 cells grown under acid adaptation (6.5) or acid recovery conditions (7.4R). The control pull-down performed with rabbit IgG is presented in Figure S3B. (**D**) Quantification of PI3K p85α subunit intensity and (**E**) CaM intensity from co-immunoprecipitation (*n* = 3–4). Welch’s correction *t*-test was used to determine the significant difference between different conditions. *, **, and *** indicate *p* < 0.05, 0.01, and 0.001, respectively. The uncropped blots are shown in File S1.

The PI3K signaling cascade is a regulator of cell cycle progression, as it activates transcription of cell cycle regulating proteins [50]. TRPC1 has been shown to be involved in activating the PI3K signaling pathway [30,33,51], and we have recently demonstrated that TRPC1 formed a complex with the PI3K p85α subunit and its associated connecting protein CaM, thereby regulating PANC-1 cell proliferation under normal pH conditions [37]. Thus, we aimed to investigate the role of the acidic tumor microenvironment and TRPC1 in association with PI3K signaling. Through confocal imaging, we found that TRPC1 also co-localized with the PI3K p85α subunit in acid-adapted PANC-1 cells, and that this was maintained under acid recovery conditions (Figure 6A). This co-localization was illustrated by the calculation of Mander’s overlapping R coefficient, which was 0.13, 0.58, and 0.48 in normal pH, acid adaptation, and acid recovery conditions, respectively (Figure 6A). We further investigated the protein interaction between the PI3K p85α subunit and TRPC1. With PLA analysis, we found an increase in the proximity between the PI3K p85α subunit and TRPC1 in acid-adapted cells by 43.8 ± 5.6% and by 28 ± 7.2% in acid-recovered cells, compared to cells grown under normal pH conditions, respectively (*n* = 3, *p* < 0.01 and 0.001, Figure 6B,C). The proximity was decreased by 21 ± 6.7% in acid-recovered cells compared to acid-adapted cells (*n* = 3, *p* < 0.01, Figure 6B,C). The protein–protein interaction was then confirmed by co-IP analysis, where we found similar results with the pull-down of both TRPC1 and PI3K p85α (Figure 6D). In addition, we investigated the interaction between CaM and TRPC1. Indeed, CaM can function as a connecting protein between TRPC1 and the p85α subunit of PI3K [51]. We found that CaM interacted with TRPC1 and the PI3K p85α subunit in all three pH conditions (Figure 6D). To strengthen the indication of a complex between TRPC1, PI3K, and CaM, we first performed PLA analysis on PANC-1 cells upon the silencing of TRPC1. We observed that the protein interaction between the PI3K p85α subunit and TRPC1 was decreased by 50.7 ± 6.2% and 34.2 ± 8.0% under acid adaptation and recovery conditions, respectively (*n* = 3, *p* < 0.001, Figure 7A,B). Next, we determined the protein–protein interaction upon the KD of TRPC1 by co-IP. Compared to siCTRL conditions, the interaction between TRPC1 and the PI3K p85α subunit was significantly decreased in siTRPC1 conditions by 79.8 ± 8.5% and 47.2 ± 14.5% in acid-adapted and -recovered cells, respectively (*n* = 4, *p* < 0.05 and 0.01, Figure 7C,D). Furthermore, a significant decrease in protein–protein interaction by 40.3 ± 10.8% and 23.0 ± 6.0% was observed between TRPC1 and CaM in acid-adapted and -recovered cells, respectively (*n* = 4, *p* < 0.05 and 0.01, Figure 7C,E). Collectively, these results indicate that TRPC1 interacts strongly with the PI3K p85α subunit under acidic and recovery conditions, probably in assembly with CaM.

### 3.6. The Knockdown of TRPC1 Decreases the Phosphorylation of AKT and ERK1/2 in Acid-Recovered PANC-1 Cells 

**Figure 8:**
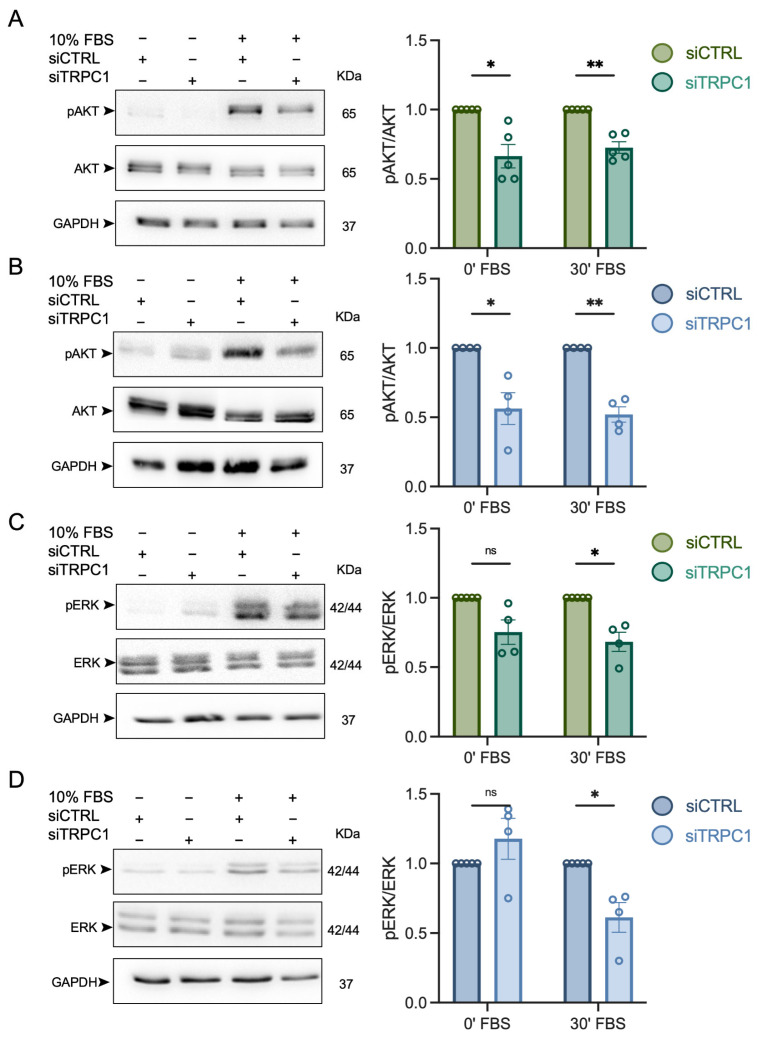
TRPC1 silencing inhibits the phosphorylation of AKT and ERK1/2, notably in acid recovery conditions. (**A**) Western blot analysis of phosphorylated AKT (pAKT) and total AKT in transfected PANC-1 cells grown in acid adaptation (6.5) conditions or (**B**) in acid recovery (7.4R) conditions after mitogen activation with FBS for either 0 or 30 min (left panel). Quantification of the Western blot analysis compared to siCTRL either after 0 min or 30 min of mitogen activation (right panel). (**C**) Western blot analysis of phosphorylated ERK1/2 (pERK1/2) and total ERK1/2 in transfected PANC-1 cells grown in acid adaptation (6.5) conditions or (**D**) in acid recovery conditions (7.4R) after mitogen activation with FBS for either 0 or 30 min (left panel). Quantification of the Western blot analysis compared to siCTRL either after 0 min or 30 min of mitogen activation (right panel). Welch’s correction *t*-test was used to determine the significant difference between siCTRL and siTRPC1. ns indicates non-significance. * and ** indicate *p* < 0.05 and 0.01, respectively. The uncropped blots are shown in File S1.

In normal pH conditions, TRPC1 silencing regulates AKT to a greater extent than ERK1/2, with a decrease of 39 ± 7.8% and 13 ± 0.4%, respectively [37]. Hence, we investigated the effect of TRPC1 silencing on the phosphorylation of AKT and ERK1/2 in acid-adapted and -recovered cells. The KD of TRPC1 reduced the activation of AKT less in acid-adapted cells (27.4 ± 4.2%), and more in acid-recovered cells (48.0 ± 5.6%), when compared to normal pH (*n* = 4–5, *p* < 0.01, Figure 8A,B). Moreover, AKT total expression remained unchanged. Silencing of TRPC1 reduced the activation of ERK1/2 strongly in acid-adapted and -recovered cells when compared to pH 7.4 (32.0 ± 6.9% and 39.0 ± 10.7%), respectively, without affecting the total ERK1/2 protein expression (*n* = 4, *p* < 0.05, Figure 8C,D). Taken together, these results indicate that TRPC1 affects the activation of AKT to a greater extent in acid-recovered PANC-1 cells and ERK1/2 phosphorylation in both conditions. 

Once we had established that TRPC1 and the PI3K p85α subunit interact with CaM, and that TRPC1 regulates AKT and ERK1/2 phosphorylation, we investigated whether the inhibition of CaM affected the downstream signaling protein kinases AKT and/or ERK1/2. Treatment of cells with the CaM inhibitor W7 for 72 h decreased the phosphorylation of AKT by 25 ± 6.8% in acid recovery conditions (*n* = 4, *p* < 0.05, Figure S4E). No significant decrease was found in normal pH or acid adaptation conditions (*n* = 3–4, Figure S4A,C). Furthermore, we found that the treatment with W7 decreased the phosphorylation of ERK1/2 by 23.8 ± 1.9%, 35.0 ± 10.5%, and 40.0 ± 6.0% in normal pH, acid adaptation, and acid recovery conditions, respectively (*n* = 4, *p* < 0.05 and 0.01, Figure S4B,D,F). These results indicate that CaM-dependent mechanisms regulate AKT solely in acid-recovered cells, while ERK1/2 levels are affected in all conditions and notably in acid-adapted and in acid-recovered PANC-1 cells.

### 3.7. PANC-1 Cell Migration and Proliferation Depend Mainly on Extracellular Ca^2+^ Entry, Likely through TRPC1, in Acid-Adapted and -Recovered PANC-1 Cells

**Figure 9:**
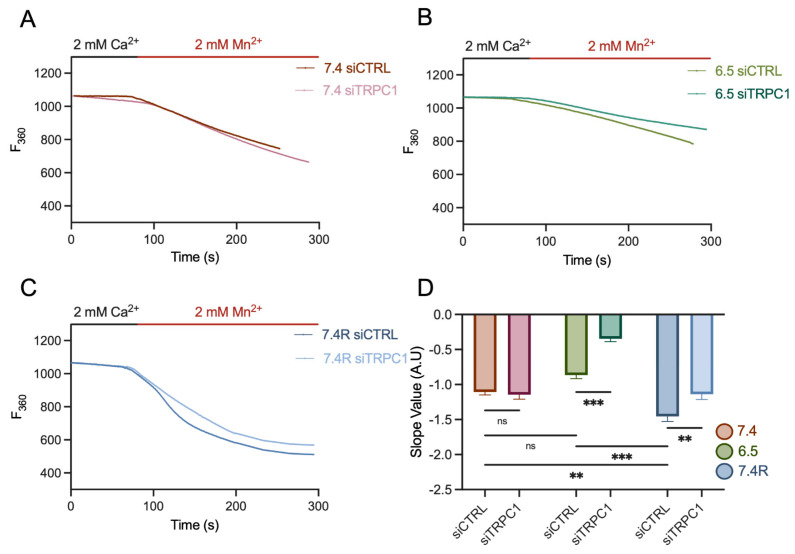
TRPC1 reduces basal Ca^2+^ entry in acid adaptation and recovery conditions. (**A**) Representative traces of Mn^2+^ quenching in transfected PANC-1 cells grown in normal pH conditions (7.4), (**B**) in acid adaptation (6.5) conditions, or (**C**) in acid recovery conditions (7.4R). (**D**) Quantification of siCTRL and siTRPC1 transfected PANC-1 cells in all three conditions (number of analyzed cells; pH 7.4 siCTRL *n* = 150 and siTRPC *n* = 128, pH 6.5 siCTRL *n* = 405 and siTRPC *n* = 326, pH 7.4R siCTRL *n* = 325 and siTRPC *n* = 214). Tukey’s multiple comparison test was used to determine significant differences between conditions. ns indicates non-significance. ** and *** indicate *p* < 0.01 and 0.001, respectively.

TRPC1 is not involved in SOCE, nor in basal Ca^2+^ entry in normal pH conditions; instead, it regulates PANC-1 cell proliferation through a Ca^2+^-independent mechanism [37]. As TRPC1 was more localized at the plasma membrane in both acid-adapted and -recovered cells compared to control, we investigated whether TRPC1 participates in Ca^2+^ entry in these conditions. First, we found an increase in Mn^2+^ quenching in acid-recovered cells (34.7 ± 5.8% and 57 ± 6.2%), compared to cells grown in normal pH and acid adaptation conditions, respectively, (*n* = 150, 405, and 325, *p* < 0.01 and 0.001, Figure 9A–D). Furthermore, we found that KD of TRPC1 decreased Mn^2+^ quenching by 60.2 ± 6.8% in acid-adapted cells (*n* = 5, *p* < 0.001, Figure 9B,D) and by 21.5 ± 6.8% in acid-recovered cells (*n* = 5, *p* < 0.01, Figure 9C,D), whereas no effect was observed in normal pH conditions (*n* = 3, Figure 9A,D). Moreover, TRPC1 KD elicited a decrease of 12.8 ± 2.4% in the basal Ca^2+^ ratio in acid-adapted cells only (*n* = 3, *p* < 0.001, Figure S5A,B), and failed to affect SOCE in acid adaptation and recovery conditions (*n* = 4, respectively, Figure S5A,C–F). 

**Figure 10:**
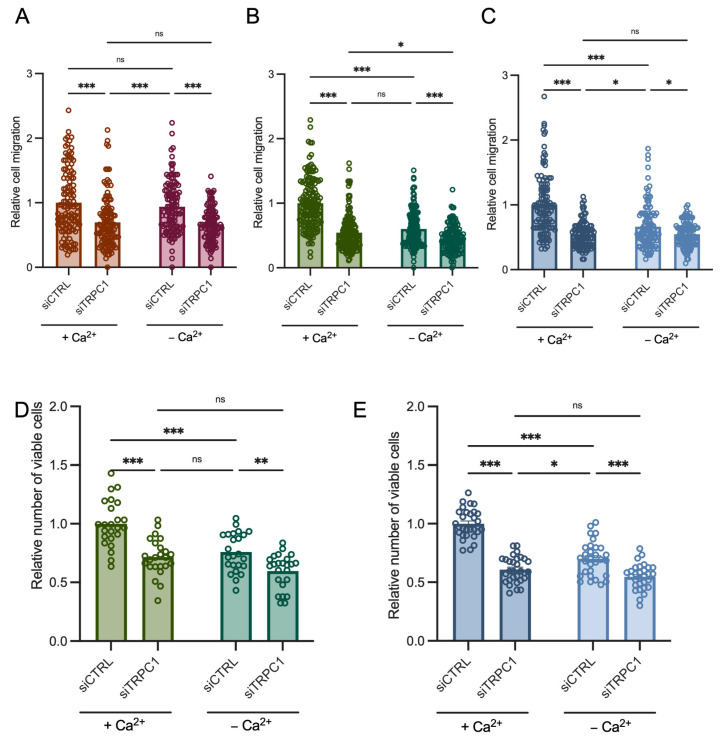
The role of TRPC1 in PANC-1 cell migration and proliferation shifts from being Ca^2+^-independent in normal pH conditions to being substantially Ca^2+^-dependent in acid adaptation and recovery conditions. (**A**) Boyden chamber assay analysis of transfected PANC-1 cells grown in normal pH conditions, (**B**) in acid-adapted (6.5) conditions, or (**C**) in acid-recovered (7.4R) conditions. Cells were transfected for 72 h in total. After 48 h, cells were seeded in Boyden inserts for 8 h, and were then treated with medium containing extracellular Ca^2+^ concentrations (+ Ca^2+^), or with a medium depleted of extracellular Ca^2+^ (− Ca^2+^), for 24 h (*n* = 3). (**D**) Trypan blue assay analysis of transfected PANC-1 cells grown in acid-adapted (6.5) conditions or (**E**) in acid-recovered (7.4R) conditions. Cells were transfected for 72 h in total and either treated with medium containing extracellular Ca^2+^ concentrations (+ Ca^2+^), or with medium depleted of extracellular Ca^2+^ (− Ca^2+^), for 48 h (*n* = 3). Tukey’s multiple comparison test was used to determine significant differences between conditions. ns indicates non-significance. *, **, and *** indicate *p* < 0.05, 0.01, and 0.001, respectively.

Given the ability of TRPC1 to regulate basal Ca^2+^ entry in acid-adapted and -recovered cells, and to understand whether Ca^2+^ is involved in cell migration and proliferation, we cultured cells in normal and low Ca^2+^ (30 µM free Ca^2+^) medium. Cell migration decreased by 40 ± 3.7% and 34.2 ± 5.6% in acid-adapted and -recovered cells, respectively, in the low Ca^2+^ medium (*n* = 3 and 4, *p* < 0.001, Figure 10B,C). KD of TRPC1 in this condition reduced migration additionally by 15.2 ± 4.7% and 12.5 ± 6.3% in acid-adapted and -recovered cells, respectively (*n* = 3 and 4, *p* < 0.001 and < 0.05, Figure 10B,C). Furthermore, no significant difference was found between the cell migration of siTRPC1 cells in normal Ca^2+^ medium and in siCTRL cells in low Ca^2+^ medium, indicating that TRPC1 mainly regulates PANC-1 cell migration in a Ca^2+^-dependent manner in acid adaptation conditions (*n* = 3, Figure 10B). In normal pH conditions, low Ca^2+^ medium failed to affect cell migration, and silencing of TRPC1 reduced it to a similar extent with or without extracellular Ca^2+^ (27.1 ± 4.7% and 30.4 ± 5.9, respectively; *n* = 3, *p* < 0.001, Figure 10A). 

Growth in low Ca^2+^ medium for 48 h decreased cell proliferation by 24.1 ± 5.3% and 30 ± 3.5% in acid adaptation and recovery conditions, respectively (*n* = 4 and 5, *p* < 0.001, Figure 10D,E). Furthermore, in low Ca^2+^ conditions, KD of TRPC1 decreased cell proliferation additionally by 16.2 ± 5.8% in acid-adapted and by 15.3 ± 4.9% in acid-recovered cells (*n* = 4 and 5, *p* < 0.01 and 0.001, Figure 10D,E). These results suggest that in acid-adapted and -recovered PANC-1 cells, proliferation and migration exhibit increased dependence on extracellular Ca^2+^ levels. TRPC1 permits Ca^2+^ entry, which regulates cell migration and proliferation by Ca^2+^-dependent and, to some extent, -independent mechanisms.

## 4. Discussion

Numerous studies have addressed the impact of TRPC1 dysregulation on hallmarks of cancer. However, the interplay between the acidic tumor microenvironment and TRPC1 expression and downstream mechanisms contributing to PDAC progression are unexplored.

The key findings of this study are: (i) The acidic tumor microenvironment promotes PDAC cell migration but attenuates proliferation, which is restored upon recovery. (ii) Acid adaptation reduces TRPC1 expression but favors its plasma membrane localization and its interaction with the PI3K p85α subunit and CaM. (iii) TRPC1 regulates acid-adapted and -recovered PANC-1 cell migration and proliferation by both Ca^2+^-dependent and -independent mechanisms. This likely occurs through the PI3K/CaM axis and the downstream activation of AKT and ERK1/2.

Acid adaptation creates an evolutionary selection pressure that contributes to a malignant phenotype [52,53]. Our results show that acid adaptation reduces PANC-1 cell viability and spheroid growth. In congruence with this, other studies have found the same events in different types of cancer cells [54,55,56], including PDAC cell lines [40,57,58]. Although acid adaptation is becoming of interest, not much is known about the recovery of and fluctuations in the acidic microenvironment. However, one study has shown that oral squamous cell carcinomas can restore their proliferation capacity after 7 and 21 days of acid treatments (pH 6.8) followed by 7 days of recovery (pH 7.4). To our knowledge, we are the first to show that 14 days of acid recovery restores PANC-1 proliferation rates, and that PANC-1 spheroid viability is enhanced after 9 days of recovery in normal pH conditions.

It is well accepted that proliferation and cell cycle progression depend on a permissive and slightly alkaline pH_i_ [41,48]. Our results show that acid-adapted PANC-1 cells exposed to pH 7.4 exhibited a more alkaline pH_i_. The acid adaptation and recovery conditions led to the accumulation of cells in G0/G1 phases and decreased the number of cells in G2/M phases. Regarding the S phase, the number of cells was increased in the acid recovery conditions, suggesting that acid adaptation promotes a more alkaline pH_i_, which results in the improved cell proliferation of acid-recovered PANC-1 cells. 

Moreover, long-term acidosis enhances extracellular matrix degradation that promotes cell migration and invasion [8,15]. Our findings are in congruence with previous reports showing that acid adaptation increased the migratory abilities of cancer cells, including PANC-1 cells [39,52,54,55,56,59]. However, the cell migration was reduced upon recovery conditions to the same levels as for normal pH conditions. Similar results were found in prostate carcinoma cells, where acute acidosis (3 h) followed by 24 h of pH 7.4 treatment decreased cell motility [60]. In oral squamous carcinoma cells, no difference in cell migration was found between acid-adapted (21 days) and -recovered (7 days) conditions. However, after only 7 days of acid treatment and 7 days of recovery under normal conditions, cell migration increased [54]. In a mouse model of metastatic breast cancer, an increase in pH to 7.4 after acid adaptation led to reduced spontaneous metastases [61]. These results suggest that migratory properties of acid-recovered cells are cell type-specific and depend on the time of acidosis and recovery.

We recently demonstrated that TRPC1 does not contribute to Ca^2+^ entry in PANC-1 cells grown under normal pH conditions [37]. Here, we show that there is a decrease in TRPC1 expression along with an increase in the plasma membrane fraction of TRPC1 in acid-adapted cells. In acid-recovered cells, the expression increased and increased plasma membrane localization of TRPC1 was maintained. In addition, TRPC1 was involved in Ca^2+^ entry in acid adaptation and recovery conditions. Moreover, the Ca^2+^ entry was increased upon acid recovery, indicating that PANC-1 cells grown in these conditions depend more on extracellular Ca^2+^ concentrations. It is unknown whether TRPC1 expression and function are affected by changes in pH. However, acidic pH activates its homologs TRPC4 and TRPC5 [35,36], leading to Ca^2+^ entry. In addition, other ion channels, transporters, and receptors can function as extracellular acid/base sensors, thereby increasing Ca^2+^ concentrations [48,62]. Furthermore, it has been reported that TRPC1 plasma membrane levels are low when in an inactive form, and that the transfer and activation of the channel depends on Ca^2+^ entry through ORAI1 and STIM1 [63,64]. Additional to other Ca^2+^ channels, TRPC1 trafficking to the plasma membrane can depend on other membrane-bound proteins. For instance, the GTP-binding protein RhoA promotes the plasma membrane translocation [65] and activation of TRPC1, which leads to SOCE [66]. The interaction between RhoA and TRPC1 leads to cell migration of intestinal epithelial cells [66]. In a glioblastoma cell line, TRPC1 translocation to the plasma membrane depends on PI3K mediated transport, which results in Ca^2+^ entry, chemotaxis, and cell migration [33]. We show that the interaction between TRPC1 and the PI3K p85α subunit/CaM was enhanced after acid adaptation. Thus, our results suggest that the acidic tumor microenvironment induces the trafficking of TRPC1 to the plasma membrane, likely in association with the PI3K p85α subunit and CaM, which are proteins near the plasma membrane. Here, TRPC1 promotes Ca^2+^ entry, in acid adaptation and recovery conditions. However, the precise mechanism of TRPC1 trafficking upon acid adaptation and recovery needs further investigation. 

TRPC1 regulates the cell cycle G1 and S phases, leading to PANC-1 cell proliferation in a Ca^2+^-independent manner [37]. In acidic and recovery conditions, we show that silencing of TRPC1 accumulated cells in the G0/G1 phases but decreased the number of cells in the G2/M phases. Remarkably, TRPC1 KD reduced the expression of CDK1, -2, -6, and cyclin A and increased the levels of p21^CIP1^ excessively in acid recovery conditions compared to normal pH and acid adaptation conditions. In addition, TRPC1 KD inhibited PANC-1 cell proliferation and spheroid growth to a greater extent than in the two other conditions. This indicates that TRPC1 expression in acid recovery conditions is of more importance in cell proliferation and spheroid growth. Furthermore, the proliferation of PANC-1 cells in acidic and recovery conditions, contrary to normal pH conditions [37], depends on extracellular Ca^2+^ levels, as the proliferation rate was decreased upon Ca^2+^ chelation. 

TRPC1 regulates the migratory properties of several cancer cell types, including PDAC cell lines [29,30,67,68,69,70]. In this study, KD of TRPC1 decreased the migratory properties of PANC-1 cells in normal, acid-adapted, and acid-recovered PANC-1 cells. However, the decrease was more prominent in acid recovery conditions. The migration of PANC-1 cells highly depends on extracellular Ca^2+^ solely in acid-adapted and -recovered cells, as cell migration decreased upon Ca^2+^ chelation. Meanwhile, no effect was found under normal pH conditions. These results indicate that, as for proliferation, TRPC1 regulates cell migration in the acidic tumor microenvironment through Ca^2+^-dependent mechanisms. 

TRPC1 forms a complex with the PI3K p85α subunit and the associated connecting protein CaM, and activates AKT and, to a lesser extent, ERK1/2 in normal pH conditions [37,51]. We report that TRPC1 strongly formed a complex with the PI3K p85α subunit, probably through CaM in acid-adapted and -recovered PANC-1 cells. This interaction was abolished upon the KD of TRPC1. Moreover, the silencing of TRPC1 resulted in a substantial decrease in AKT and ERK1/2 activation in acid-adapted and -recovered cells, which could result in the downregulation of cell-cycle-regulating proteins and, thereby, cell cycle arrest in the G0/G1 and G2/M phases. These results indicate that in these conditions, TRPC1 regulates proliferation and migration both through the PI3K and MAPK signaling axis, and that TRPC1 exerts a more aggressive role in acid-recovered PANC-1 cells. 

AKT and ERK1/2 have previously been shown to be regulated through TRPC1 by Ca^2+^-dependent and -independent mechanisms. The activation of AKT depends on Ca^2+^ through TRPC1 in lung cancer and hepatocellular carcinoma cell lines [71,72], and the activation of ERK1/2 depends on Ca^2+^ through TRPC1 in thyroid and different breast cancer cell lines [29,73,74]. Here, the activation of AKT and ERK1/2 seemed to be regulated, at least partially, by Ca^2+^ through TRPC1 in acid adaptation and recovery conditions. As TRPC1 forms a complex with PI3K p85α subunit and the Ca^2+^ binding messenger CaM, we investigated whether the inhibition of CaM affected the activation of AKT and ERK1/2. This inhibition only decreased the activating phosphorylation of AKT in acid recovery conditions, where the activation of ERK1/2 was reduced in all conditions, but notably in acid-adapted and -recovered cells. Our results designate the importance of CaM and Ca^2+^ downstream signaling in acid recovery conditions.

Interestingly, in more than 90% of PDAC tumors, the oncogene *KRAS* is mutated, and *KRAS* G12D is the predominant driver mutation in this type of adenocarcinoma [75]. Furthermore, *KRAS* is involved in reprogramming cell metabolism, including glucose metabolism, and the mutations are known to promote aerobic glycolysis (also known as the Warburg effect) [76]. The change in aerobic glycolysis in cancer cells is well known to produce waste products in the form of H^+^, causing increased proton extrusion and an acidic extracellular space. As TRPC1 can potentially regulate MAPK signaling cascades through *KRAS* in liver and colorectal cancer cells lines [51,77], it would be interesting to further investigate the interplay between TRPC1, *KRAS*, and the metabolic changes implicated in the acidification of the tumor microenvironment of PDAC. 

## 5. Conclusions

**Figure 11:**
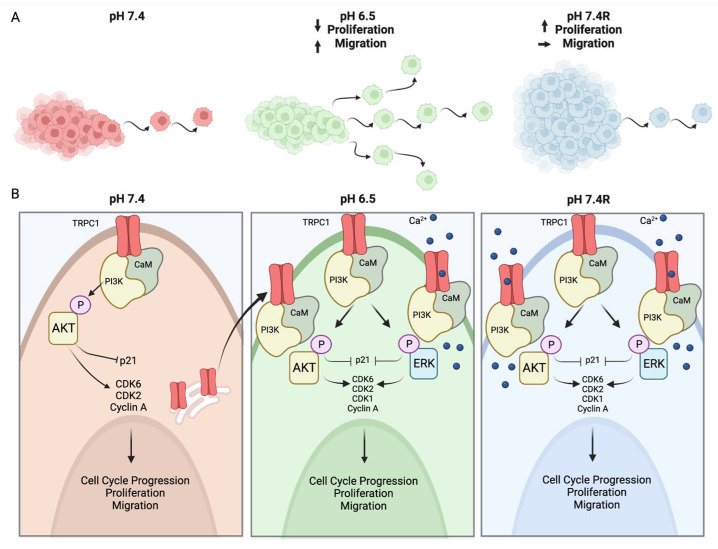
(**A**) Acid adaptation promotes migration but attenuates proliferation, whereas acid recovery impairs this migration but further accelerates proliferation. (**B**) The fluctuations in the acidic tumor microenvironment affect TRPC1 expression and localization. TRPC1 is downregulated in acid-adapted PANC-1 cells and favors plasma membrane localization, which is maintained in acid-recovered PANC-1 cells, where the expression of TRPC1 is upregulated. In the plasma membrane of acid-adapted and -recovered cells, TRPC1 permits Ca^2+^ entry and, in interactions with the PI3K p85α subunit and CaM, regulates PANC-1 cell migration, proliferation, and cell cycle progression. The figure is generated with www.Biorender.com.

In conclusion, we show that pH fluctuations in the tumor microenvironment affect PDAC cell migration, proliferation, and spheroid growth. We demonstrate that acid adaptation permits TRPC1 localization to the plasma membrane. Here, TRPC1 reinforces a complex with PI3K p85α/CaM and promotes Ca^2+^ entry. Synergically, this regulates AKT and ERK1/2 activation, which in turn controls cell cycle progression, proliferation, and migration. This indicates that the fluctuations in the acidic tumor microenvironment confer a more aggressive role to TRPC1 (Figure 11). 

## Data Availability

The data presented in this study are available in this article and Supplementary Material.

## Supplementary Materials

The following supporting information can be downloaded at: https://www.mdpi.com/article/10.3390/cancers14194946/s1. Figure S1: Knockdown of TRPC1 in 2D and 3D models evaluated by qPCR and WB. Figure S2: Trypan blue assay performed in parallel with Boyden chamber assay to avoid any bias due to cell proliferation. Annexin-5 assay shows no effect upon siTRPC1. Figure S3: Representative WB of non-affected cell cycle regulating proteins upon the KD of TRPC1 and of the control pull-down with IgG-Rabbit for co-IP. Figure S4: Representative WB of PANC-1 cells treated with W7, blotted for pERK, ERK, pAKT and AKT. Figure S5: Representative traces of the classical store-operated Ca^2+^ entry protocol [78]. File S1. Full pictures of the Western blots.
