# Supplementary material for: Acid Adaptation Promotes TRPC1 Plasma Membrane Localization Leading to Pancreatic Ductal Adenocarcinoma Cell Proliferation and Migration through Ca2+ Entry and Interaction with PI3K/CaM
Source: Cancers (Basel). 2022 Oct 9;14(19):4946. doi: 10.3390/cancers14194946 (PMC9563726; doi:10.3390/cancers14194946)
Supplement: Supplementary file 1 [file cancers-14-04946-s001.zip › cancers-1887190-supplementary.pdf]

cancers

MDPI

Supplementary Figures and Material and Methods

Acid Adaptation Promotes TRPC1 Plasma Membrane Localization Leading to Pancreatic Ductal Adenocarcinoma Cell Proliferation and Migration through Ca^{2+} Entry and Interaction with PI3K/CaM

Julie Schnipper [1], Sana Kouba [1], Frédéric Hague [1], Alban Girault [1], Marie-Sophie Telliez [1], Stéphanie Guénin [2], Ahmed Ahidouch [1,3], Stine Falsig Pedersen [4] and Halima Ouadid-Ahidouch [1,*]

[1] Laboratory of Cellular and Molecular Physiology, UR UPJV 4667, University of Picardie Jules Verne, 80000 Amiens, France
[2] Regional Ressources Center for Molecular Biology (CRRBM), University of Picardie Jules Verne, 80000 Amiens, France
[3] Biology Department, Sciences Faculty, University Ibn Zohr, Agadir 80000, Morocco
[4] Section for Cell Biology and Physiology, Department of Biology, University of Copenhagen, 2100 Copenhagen Ø, Denmark
* Correspondence: halima.ahidouch-ouadid@u-picardie.fr; Tel.: +33-322-827-646

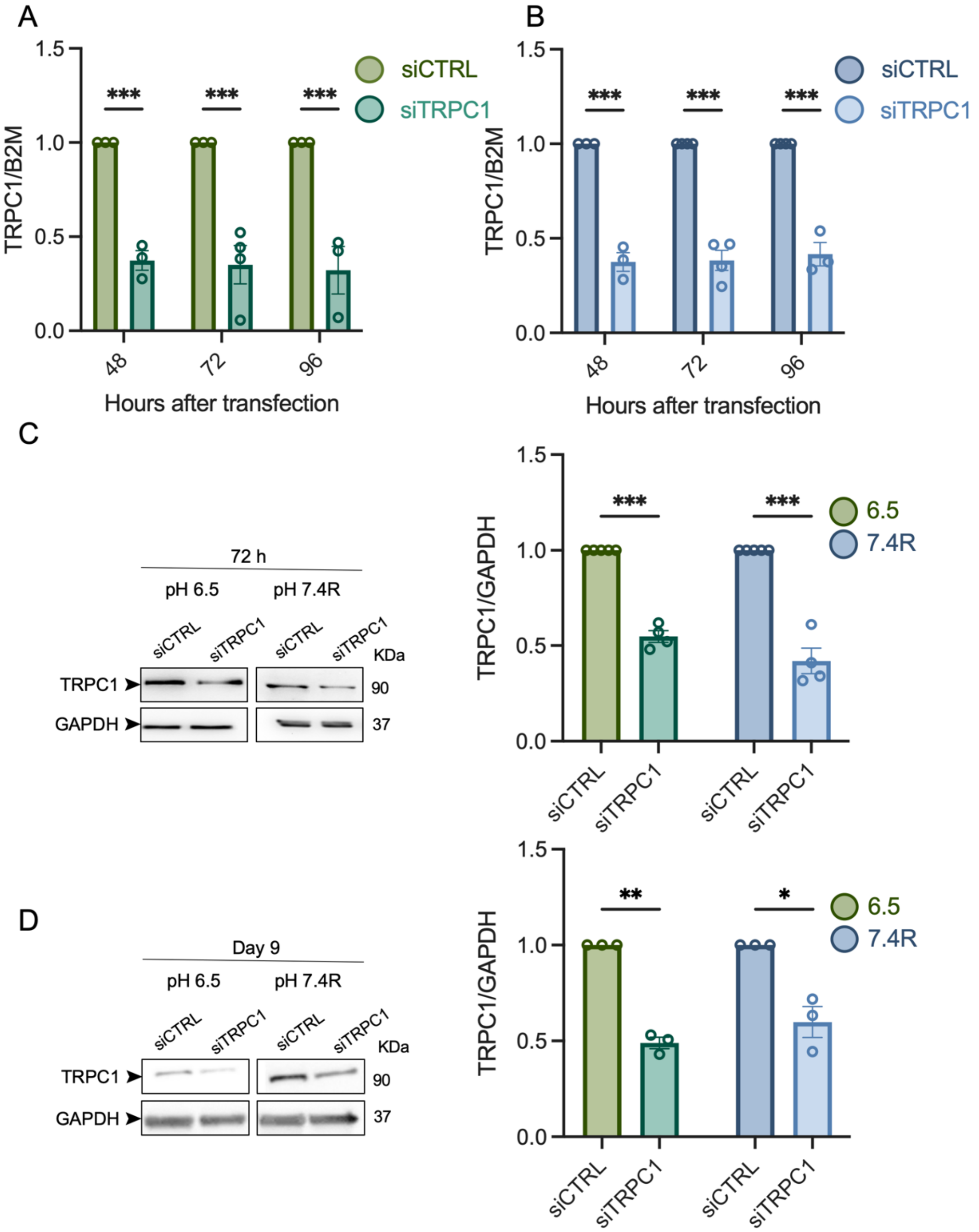

Figure S1. (**A**) TRPC1 mRNA expression evaluated by qPCR 48, 72 and 96 h after transfection with siCTRL and siTRPC1 in acid-adapted PANC-1 cells (6.5) (n = 3-4). (**B**) TRPC1 mRNA expression evaluated by qPCR 48, 72 and 96 h after transfection with siCTRL and siTRPC1 in acid-recovered PANC-1 cells (7.4R) (n = 3-4). Materials and methods for qPCR are described below. (**C**) TRPC1 protein expression evaluated 72 h after transfection with siCTRL and siTRPC1 in acid-adapted (6.5) or acid-recovered (7.4R) PANC-1 cells (n = 4, Western blot analysis left panel and quantification right panel). (**D**) TRPC1 protein expression evaluated 9 days after transfection with siCTRL and siTRPC1 in acid-adapted PANC-1 spheroids, grown in medium pH 6.5 or 7.4R (n = 3, Western blot analysis left panel and quantification right panel). Welch's correction t-test was used to determine the significant difference between siCTRL and siTRPC1 conditions. *, ** and *** indicate $p < 0.05$, 0.01 and 0.001, respectively.

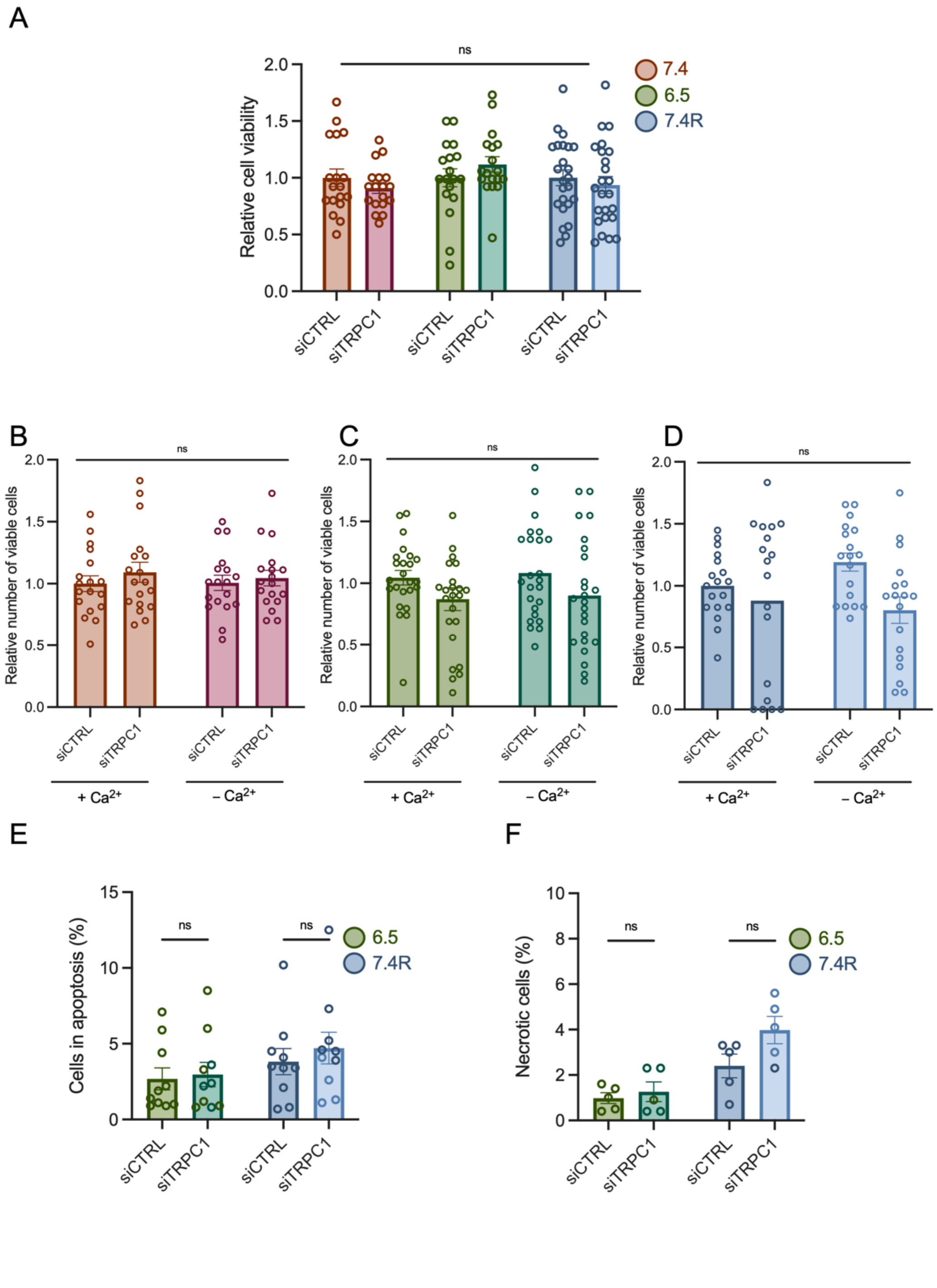

Figure S2. (**A**) Trypan blue assay performed in parallel with Boyden chamber assay (24 h after seeding). Welch's correction t-test was used to determine the significant difference between siCTRL and siTRPC1 conditions (n = 3-4). Ns indicates non-significant. (**B**) Trypan blue assay performed in parallel with Boyden chamber assay, with (+ Ca^{2+}) or without (- Ca^{2+}) calcium for 24 h in PANC-1 cells grown under normal pH conditions (7.4), (n = 3) (**C**) in acid-adapted (n = 4) or, (**D**) acid-recovered conditions (n = 3). Tukey's multiple comparison test was used to determine significant differences between conditions. Ns indicates non-significant. (**E**) Quantification of Annexin-5 analysis of cells in apoptosis or, (**F**) necrotic cells 72 h after transfection with siCTRL and siTRPC1 in acid-adapted (6.5) or acid-recovered (7.4R) PANC-1 cells (n = 3). Welch's correction t-test was used to determine the significant differences between siCTRL and siTRPC1 conditions. Ns indicates non-significant. Materials and methods for Annexin-5 analysis are described below.

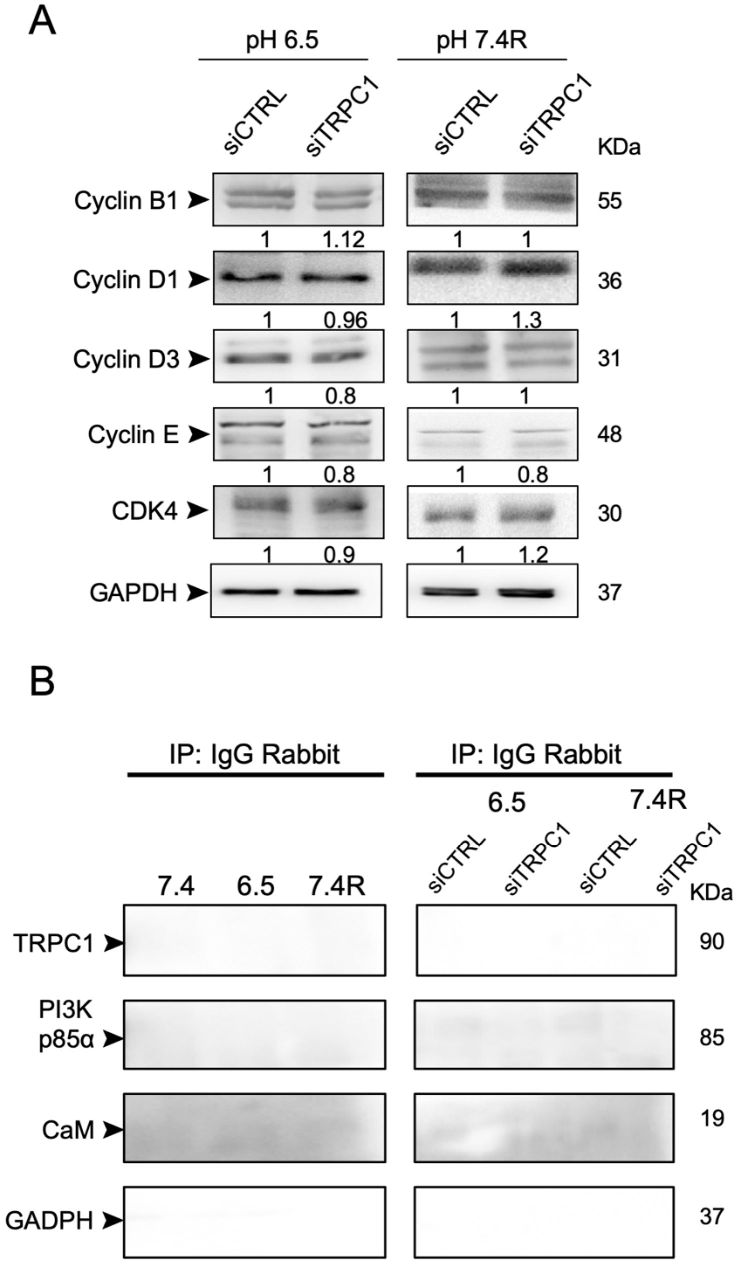

Figure S3. (**A**) Westernblot analysis of different cell cycle regulating proteins 72 h after transfection with siCTRL and siTRPC1 in acid-adapted (6.5) or acid-recovered (7.4R) PANC-1 cells (*n*= 3-4, no significance was found by using Welch's correction t-test). (**B**) Representative immunoblot of co-immunoprecipitation with a control IgG Rabbit.

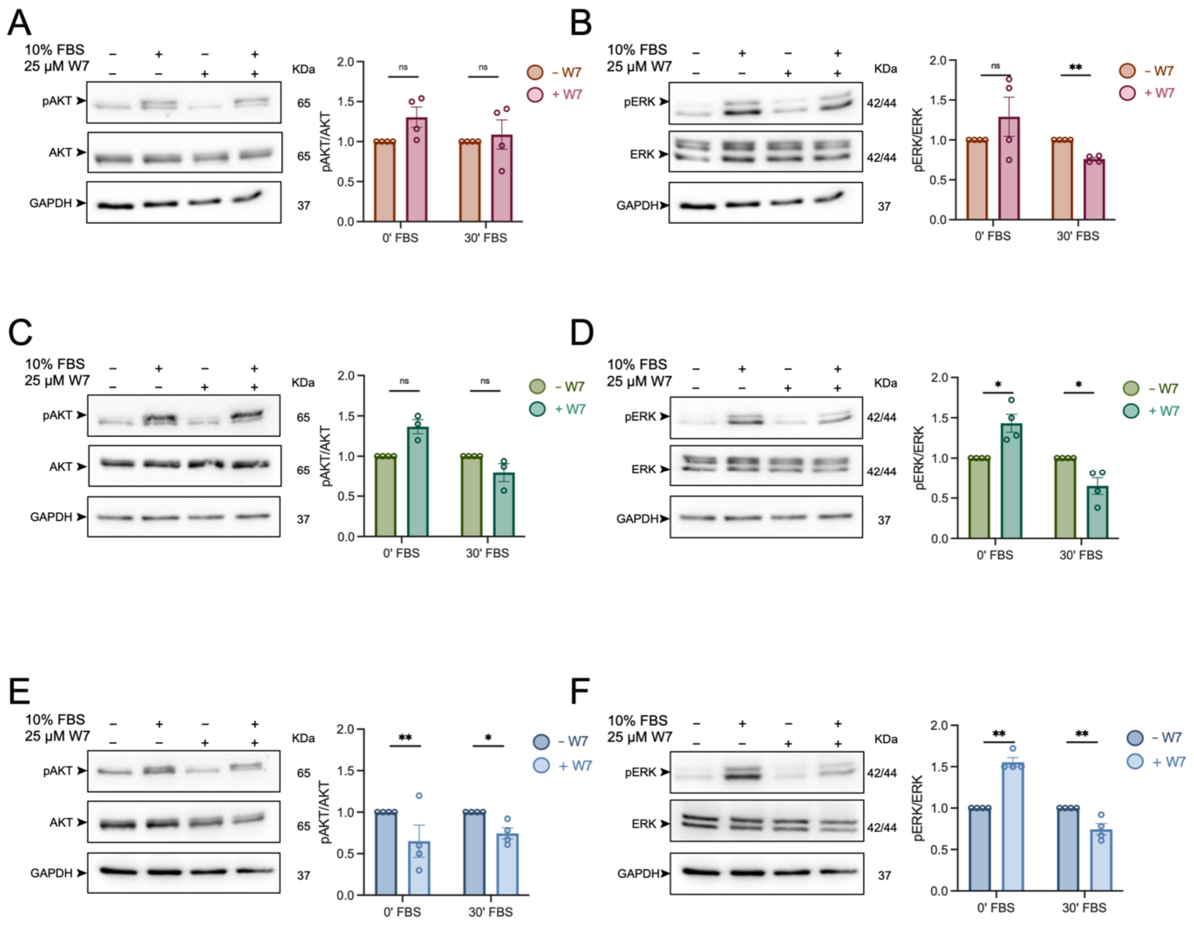

Figure S4. Representative immunoblot analysis of PANC-1 cells treated with 72 h of 25 µM W7, including 24 h of starvation, followed by either 0 min or 30 min of mitogen activation with 10% FBS. (**A**) phosphorlyated AKT (pAKT) and total AKT in normal conditions (7.4). (**B**) phosphorlyated ERK1/2 (pERK) and total ERK1/2 in normal conditions (7.4). (**C**) phosphorlyated AKT (pAKT) and total AKT in acid-adapted conditions (6.5). (**D**) phosphorlyated ERK1/2 (pERK) and total ERK1/2 in acid-adapted conditions (6.5). (**E**) phosphorlyated AKT (pAKT) and total AKT in acid-recovered conditions (7.4R). (**F**) phosphorlyated ERK1/2 (pERK) and total ERK1/2 in acid-recovered conditions (7.4R). (n = 3-4). Welch's correction t-test was used to determine the significant difference between conditions. Ns indicates non-significant. * and ** indicate $p < 0.05$ and 0.01, respectively.

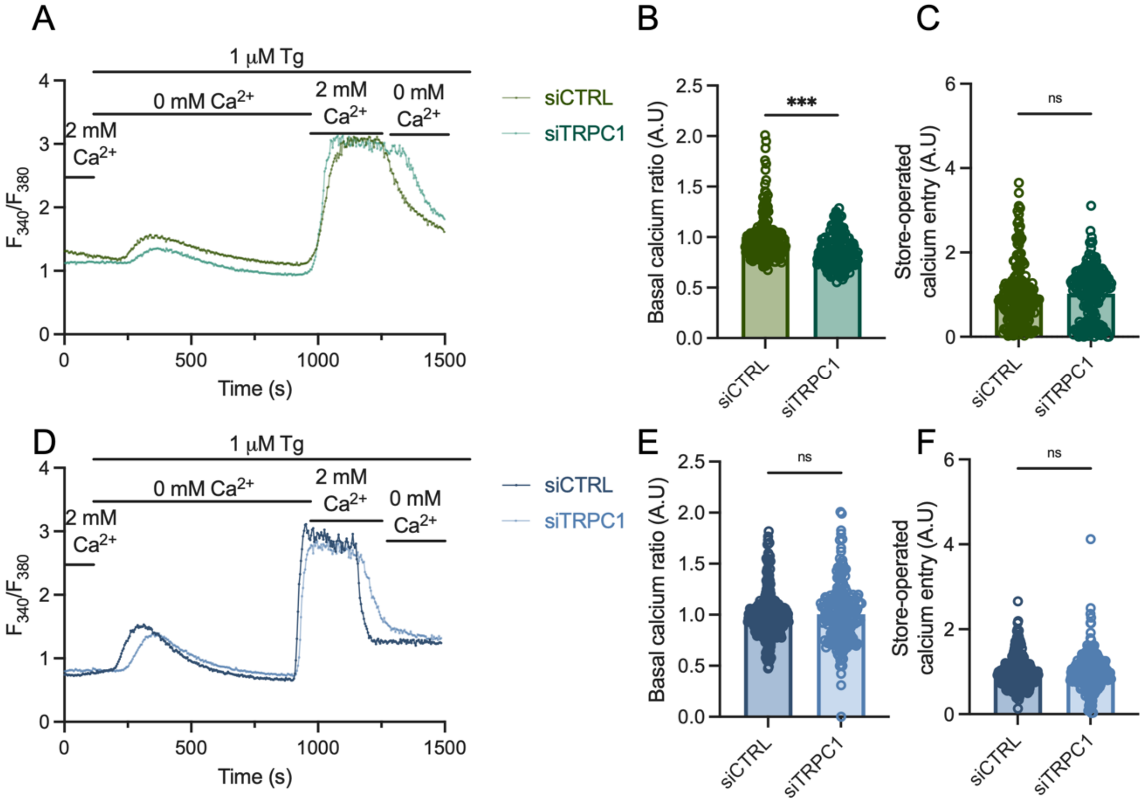

Figure S5. (**A**) Representative traces of the classical store-operated Ca^{2+} entry protocol. Acid-adapted (6.5) PANC-1 cells were perfused with 2mM Ca^{2+} for 1 min, then with 0 mM Ca^{2+} and 1 μM thapsigargin (Tg) for 12 min, followed by 2 mM Ca^{2+} for 5 min, and finally perfused with 0 mM Ca^{2+}. (**B**) Quantification of basal Ca^{2+} ratio (0 mM Ca^{2+}) and (**C**) quantification of SOCE (2 mM Ca^{2} after internal Ca^{2+}-store depletion) in in acid-adapted (6.5) and transfected PANC-1 cells (number of analyzed cells siCTRL = 183 and siTRPC1 = 158). (**D**) Representative traces of the classical store-operated Ca^{2+} entry protocol used on acid-recovered (7.4R) PANC-1 cells. (**E**) Quantification of basal Ca^{2+} ratio (0 mM Ca^{2+}) and (**F**) quantification of SOCE (2 mM Ca^{2} after internal Ca^{2+}-store depletion) in acid-recovered (7.4R) and transfected PANC-1 cells (number of analyzed cells siCTRL = 274 and siTRPC1 = 234). Welch's correction t-test was used to determine the significant difference between conditions. Ns indicates non-significant. * indicates $p < 0.05$.

Supplemental Materials and Methods

Store-operated Ca^{2+} entry assay

To investigate Store-operated Ca^{2+} entry (SOCE) we used the same equipment and reagents as described in the Mn^{2+} Quench Assay. SOCE was triggered by applying the classical protocol, first by perfusing a saline solution (145 mM NaCl, 5 mM KCl, 2 mM $CaCl_2$, 1 mM $MgCl_2$, 5 mM glucose, 10 mM HEPES, pH 7.4 or pH 6.5) containing 2 mM Ca^{2+} (for 1 min), then 0 mM Ca^{2+} during 1 min followed by 1 μM thapsigargin in 0 mM Ca^{2+} for 12 min to induce Ca^{2+} store depletion, followed by perfusion of 2 mM Ca^{2+} (for 5 min). The intracellular Ca^{2+} concentration is derived from the ratio of emitted fluorescence intensities for each of the excitation wavelengths (F340/F380), and emission at 510 nm.

Quantitative Real-Time PCR (qRT‐PCR)

The 2.5 × 10^4 transfected (with either siCTRL or siTRPC1) PANC-1 cells, grown in either pH 6.5 or 7.4R conditions, were seeded for 48, 72, or 96 h respectively, to determine the KD of TRPC1 at the transcriptional level. At the specific time points, total RNA was extracted with the Trizol reagent (Sigma-Aldrich, Saint-Quentin-Fallavier, France) method as previously described [44,47]. After RNA extraction, RNA concentrations and purity were determined using a spectrophotometer (NanoDrop 2000, Wilmington, NC, USA). 2 μg of RNA was converted into cDNA with the MultiScribe™ Reverse Transcriptase kit (Applied Biosystems, Carlsbad, CA, USA). Real‐time PCR was performed on a LightCycler 480 System (Roche, Basel, Switzerland) using SYBR Green I PCR master mix (Life Science, Roche, Basel, Switzerland). Sense and antisense PCR primers specific to TRPC1 (forward 5′ GAGGTGATGGCGCTGAAGG-3′ and reverse 5′‐GCACGCCAGCAAGAAAAGC-3′) and β2 microglobulin (B2M) (forward 5′GTCTTTCAGCAAGGACTGGTC-3’ and reverse CAAATGCGGCATCTTCAAACC3′). TRPC1 mRNA expression was normalized to B2M, used as housekeeping gene, and compared to the control sample, using the Pfaffl method [78].

FACS analysis of annexin V and propidium iodide staining

To evaluate the percentage of apoptotic and necrotic cells, we detected phosphatidylserine residues at the outer plasma membrane by the FITC Annexin V Apoptosis Detection Kit I (BD Biosciences Pharmingen, Le Pont‐de‐Claix, France). 5 × 10^5 transfected PANC‐1 cells, grown in either pH 6.5 or 7.4R conditions, were seeded and collected after 72 h. Both detached and adherent cells were collected by trypsinization. Cells were pelleted, washed twice with cold PBS and resuspended in 1X binding buffer (BD Biosciences Pharmingen). Following the Annexin V Apoptosis Detection Kit staining protocol, we added FITC Annexin V and propidium iodide to the cell pellets and incubated them at room temperature in the dark for 15 min. The binding buffer was then added to each sample, which were immediately analyzed by flow cytometry (Accuri®) in order to determine the percentage of apoptotic and necrotic cells.
