# Supplementary material for: Acid Adaptation Promotes TRPC1 Plasma Membrane Localization Leading to Pancreatic Ductal Adenocarcinoma Cell Proliferation and Migration through Ca2+ Entry and Interaction with PI3K/CaM
Source: Cancers (Basel). 2022 Oct 9;14(19):4946. doi: 10.3390/cancers14194946 (PMC9563726; doi:10.3390/cancers14194946)

Figure 1

Fig. 1B
TRPC1 90 KDa

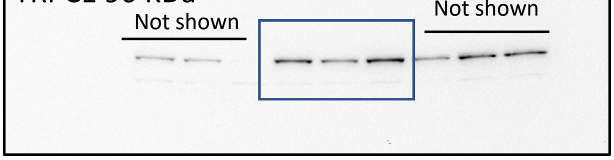

Fig. 1B
GAPDH 37 KDa

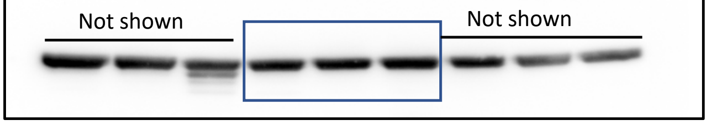

Fig. 1C
TRPC1 90 KDa

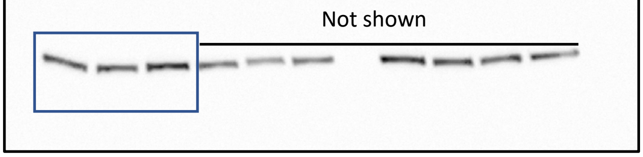

Fig. 1C
GAPDH 37 KDa

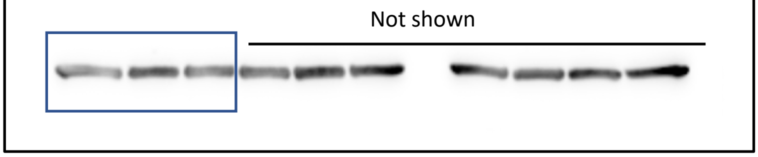

Fig. 1E Membrane fraction
TRPC1 90 KDa

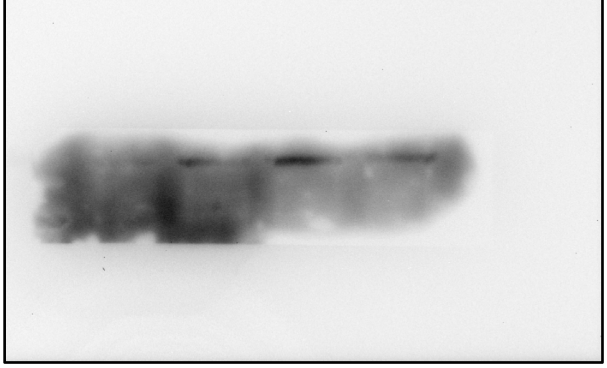

Fig. 1E Total Lysate
TRPC1 90 KDa

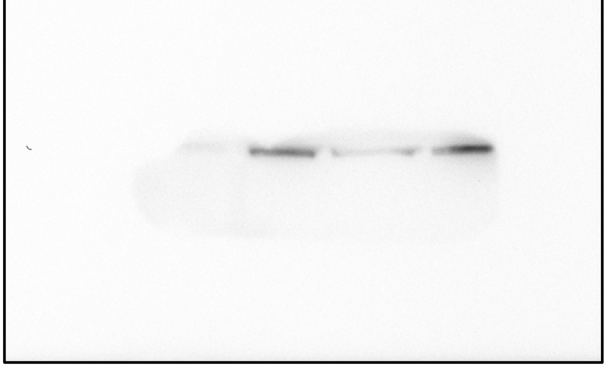

Fig. 1E
GAPDH 37 KDa

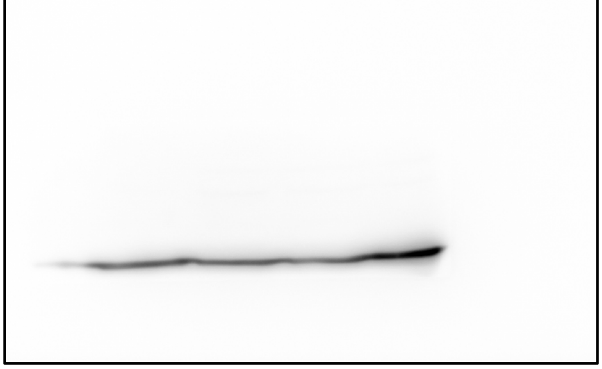

Supplemental Figure S1

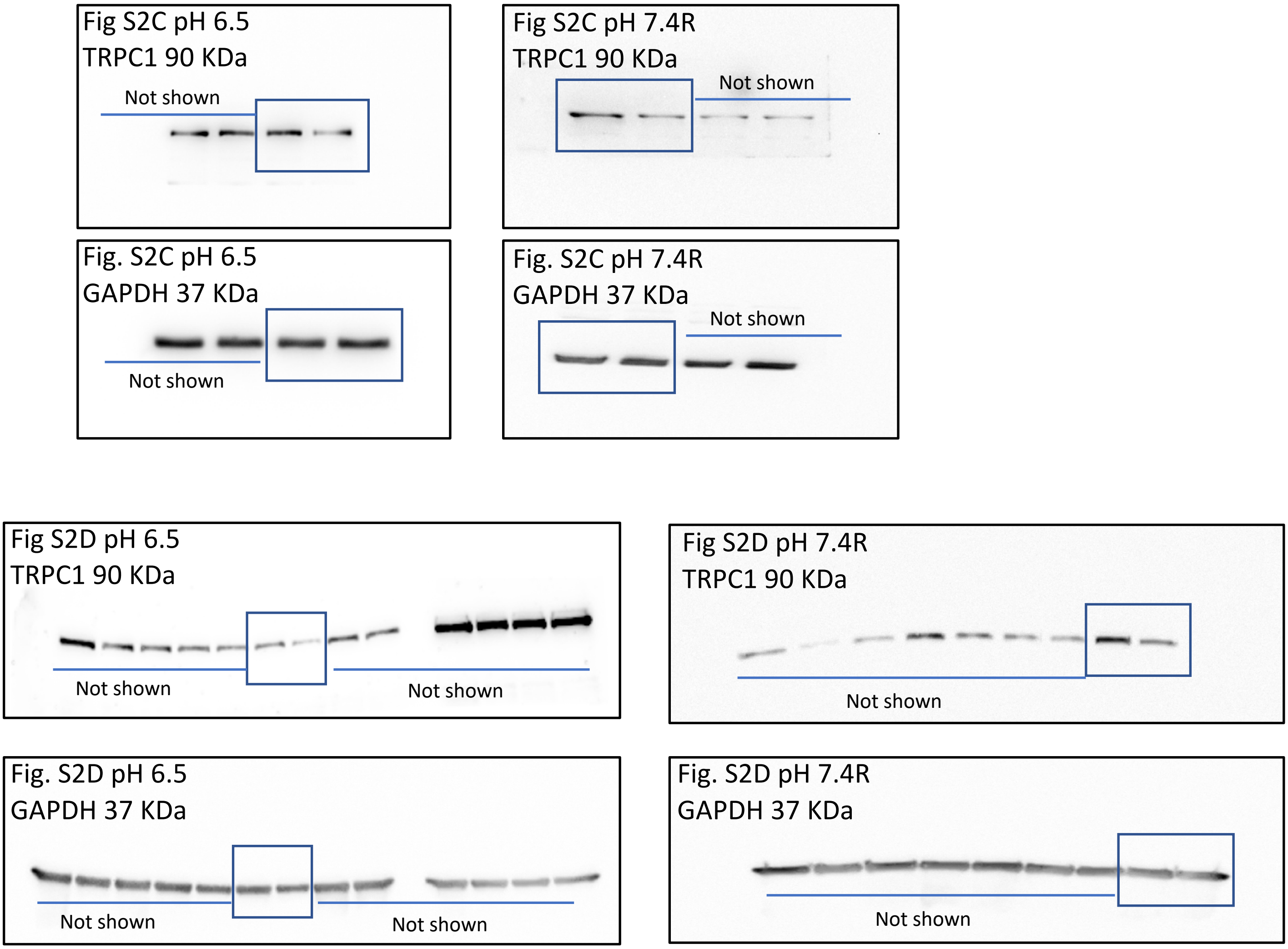

Figure 5A

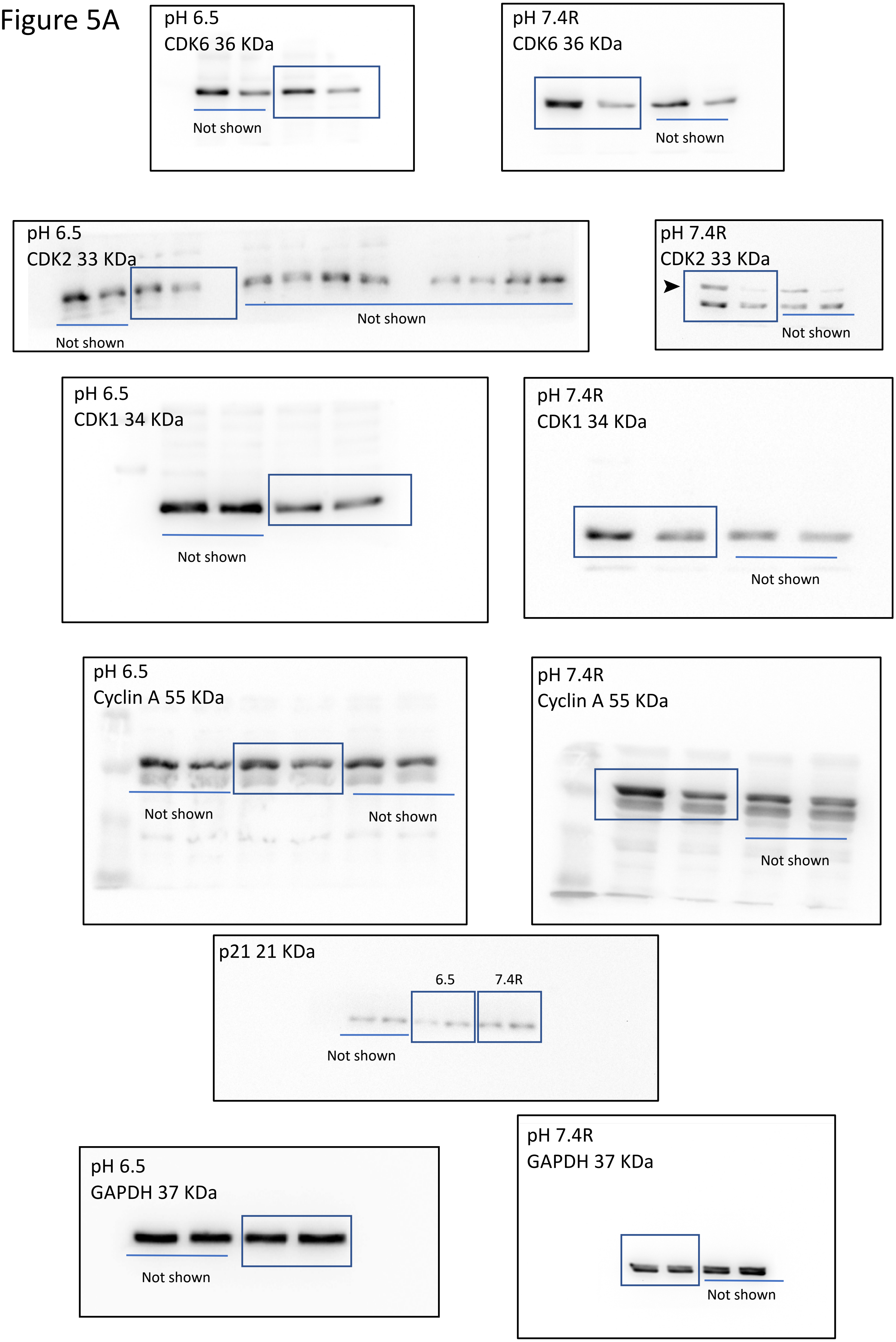
pH 6.5
CDK6 36 KDa
Not shown
pH 7.4R
CDK6 36 KDa
Not shown
pH 6.5
CDK2 33 KDa
Not shown
Not shown
pH 7.4R
CDK2 33 KDa
Not shown
pH 6.5
CDK1 34 KDa
Not shown
pH 7.4R
CDK1 34 KDa
Not shown
pH 6.5
Cyclin A 55 KDa
Not shown
Not shown
pH 7.4R
Cyclin A 55 KDa
Not shown
p21 21 KDa
6.5
7.4R
Not shown
pH 6.5
GAPDH 37 KDa
Not shown
pH 7.4R
GAPDH 37 KDa
Not shown

Supplemental Figure S3A

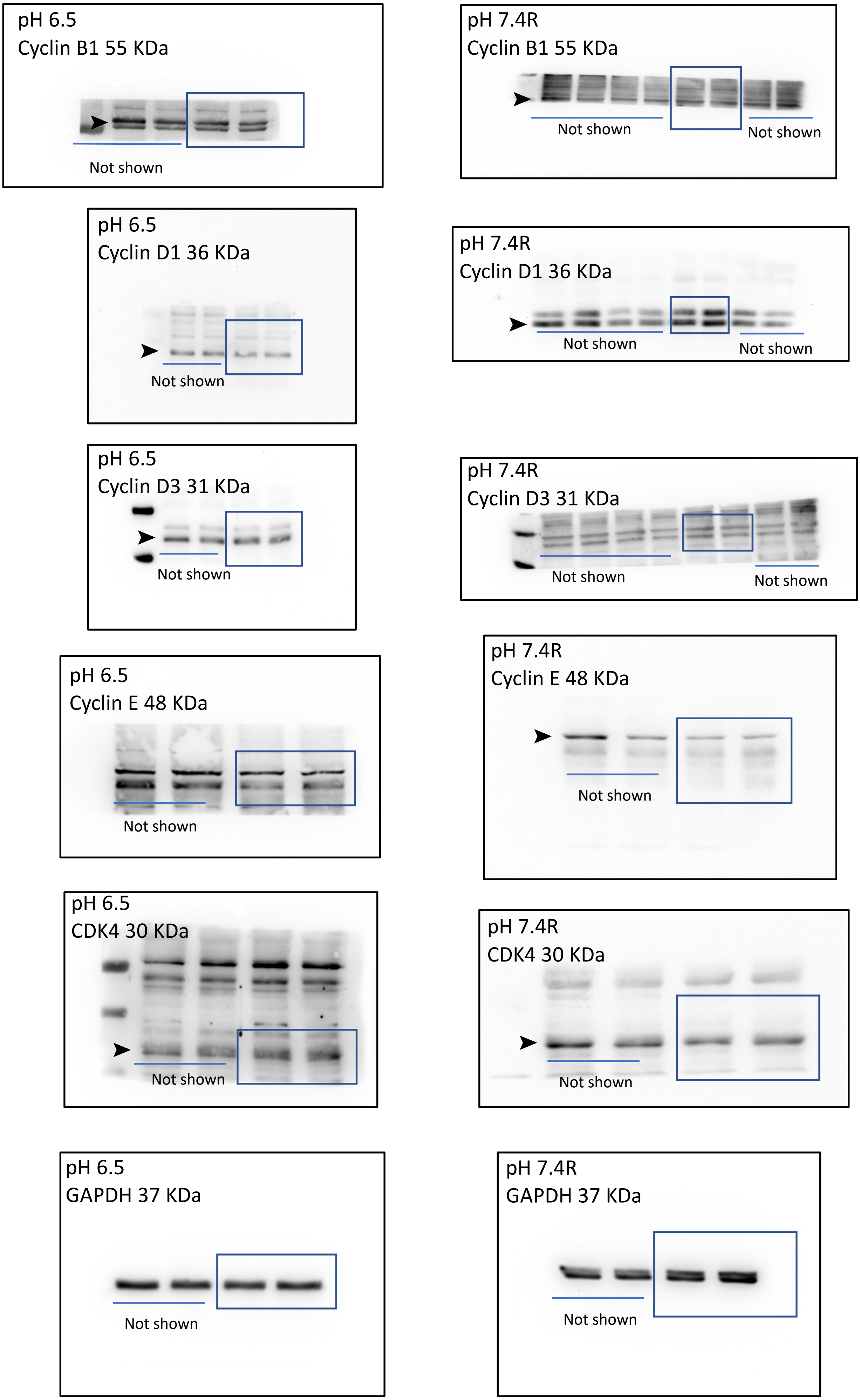

Supplemental Figure S3B

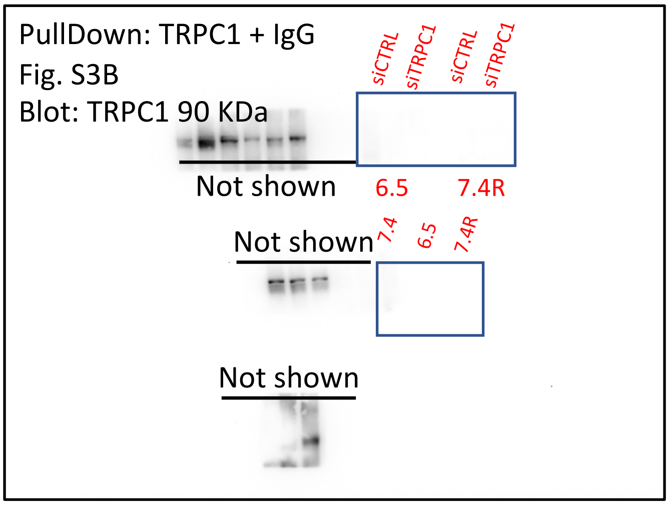

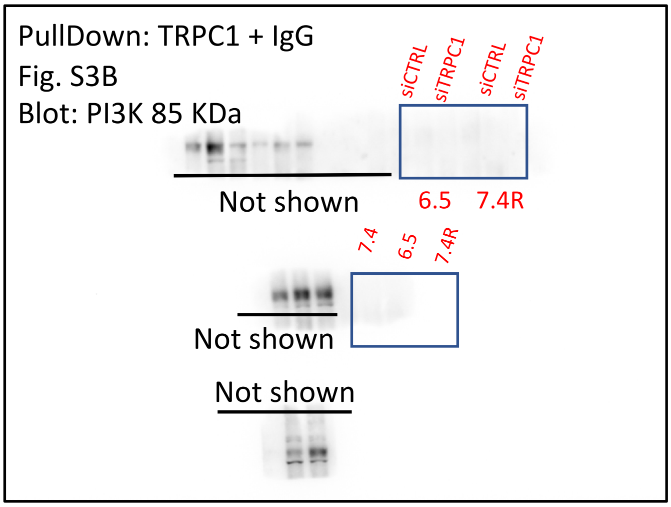

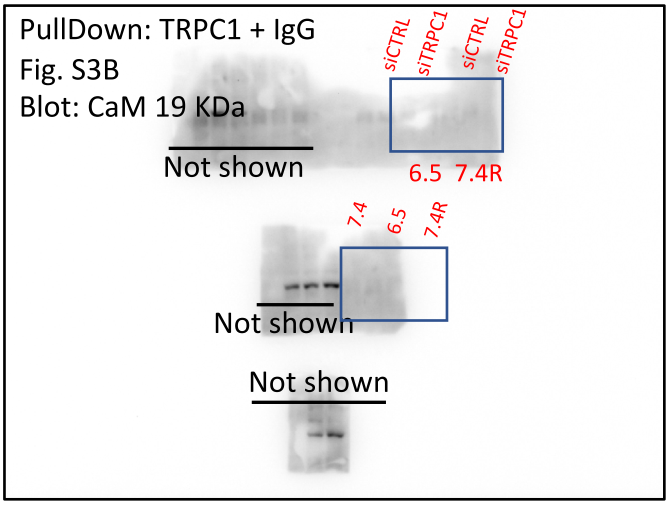

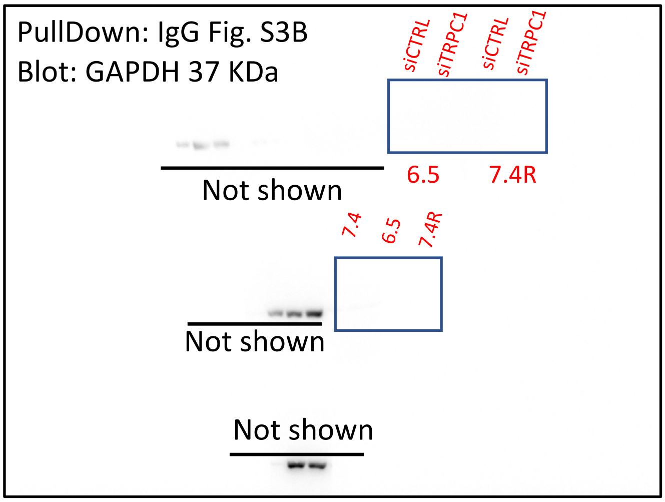

Figure 6D

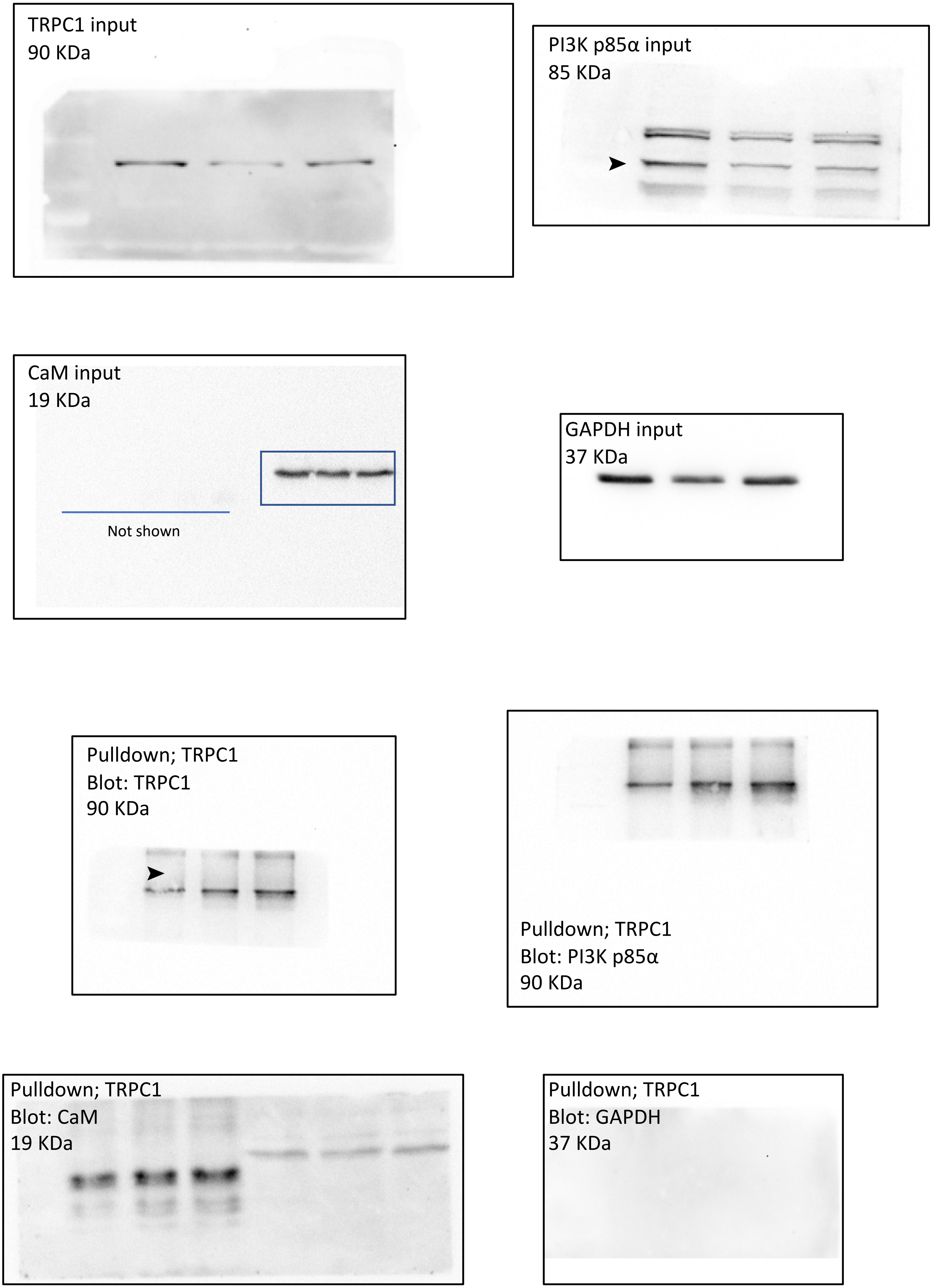
TRPC1 input
90 KDa
PI3K p85α input
85 KDa
CaM input
19 KDa
Not shown
GAPDH input
37 KDa
Pulldown; TRPC1
Blot: TRPC1
90 KDa
Pulldown; TRPC1
Blot: PI3K p85α
90 KDa
Pulldown; TRPC1
Blot: CaM
19 KDa
Pulldown; TRPC1
Blot: GAPDH
37 KDa

Figure 6D

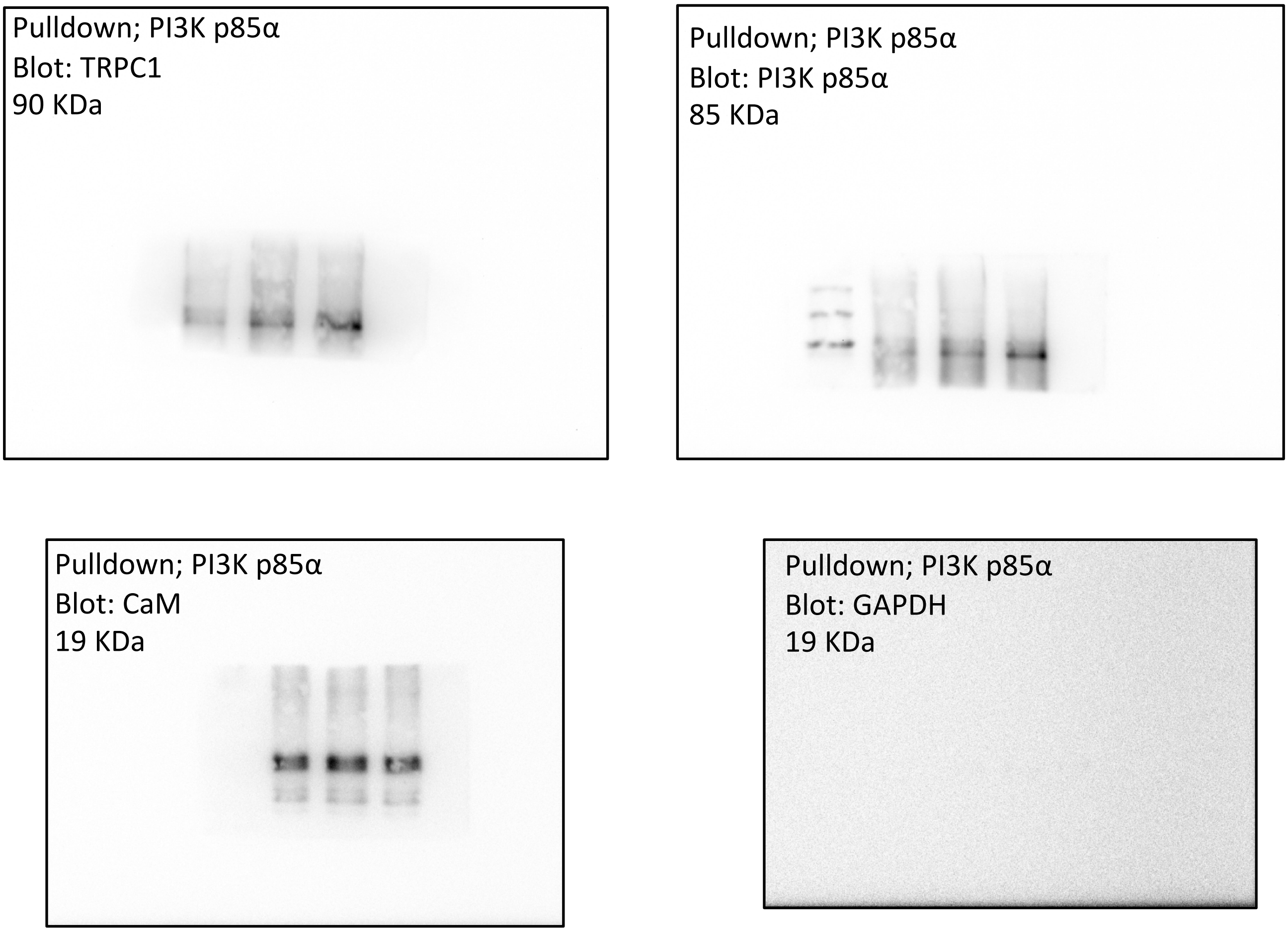

Figure 7C pH 6.5

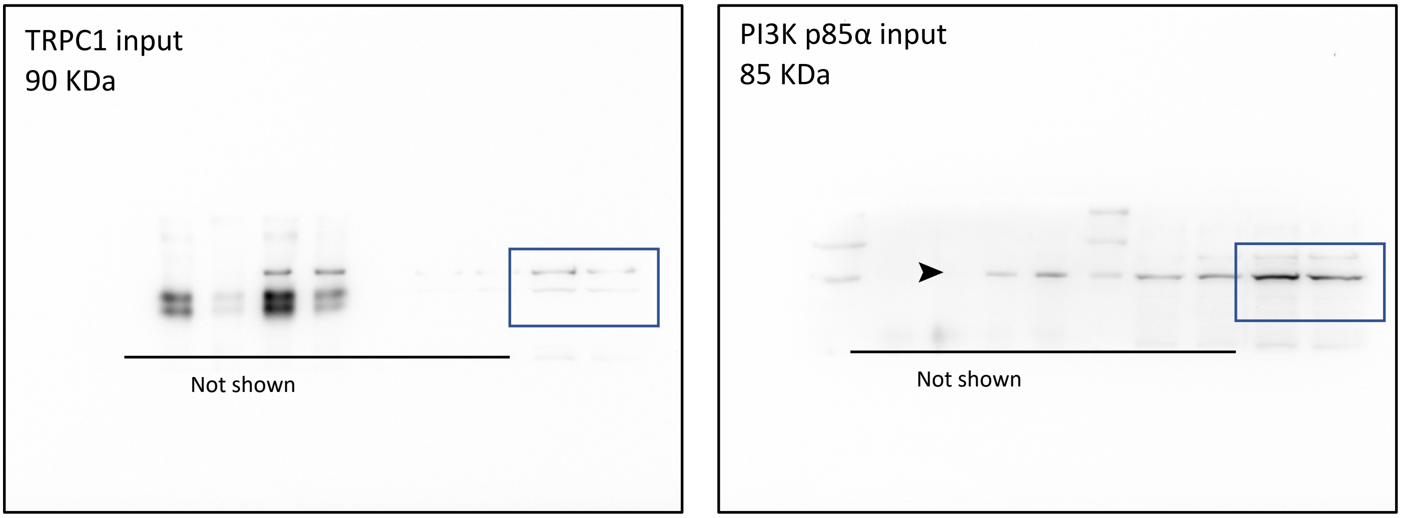

Figure 7C pH 6.5

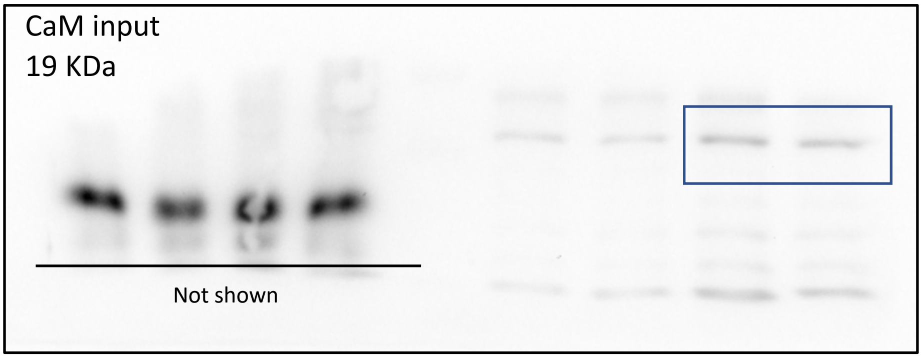

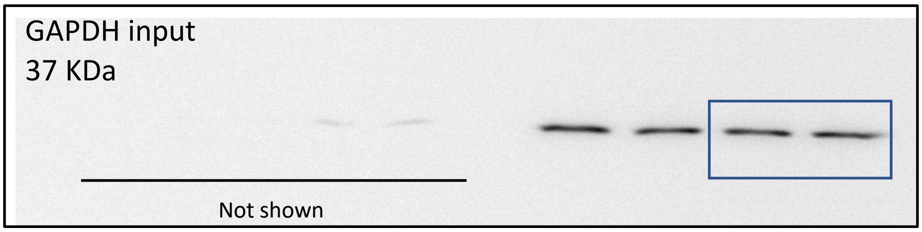

Figure 7C pH 7.4R

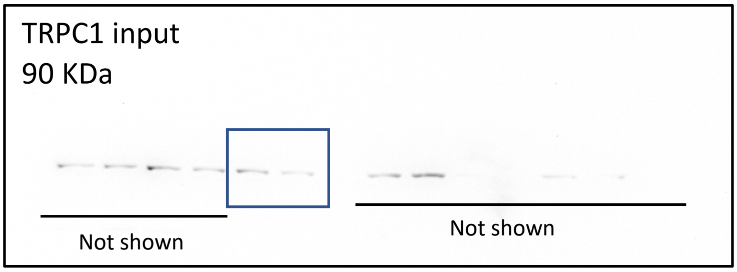

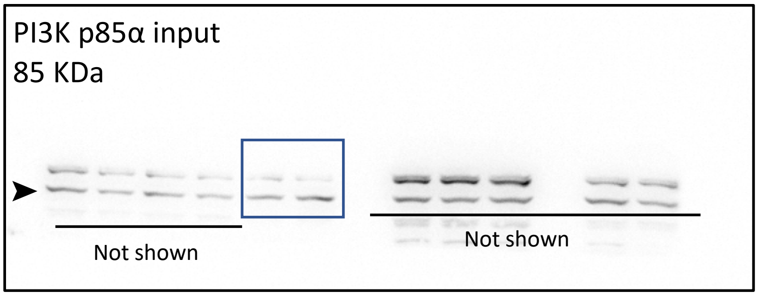

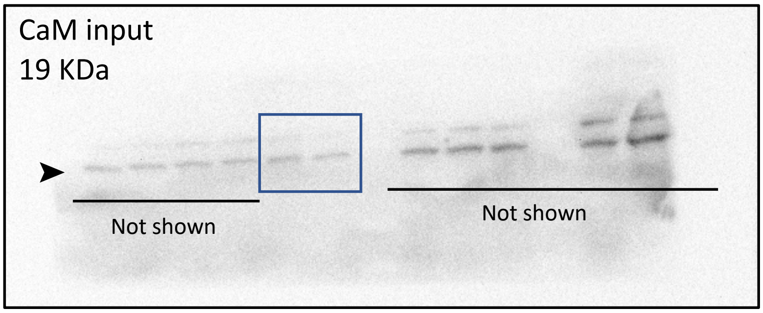

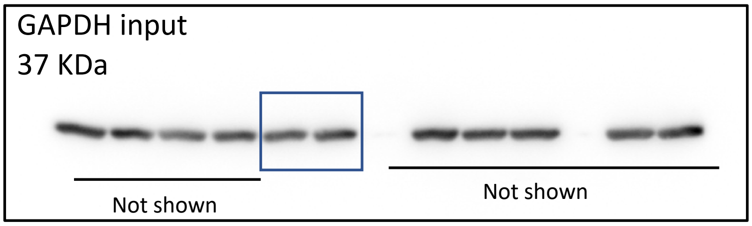

Figure 7C pH 6.5

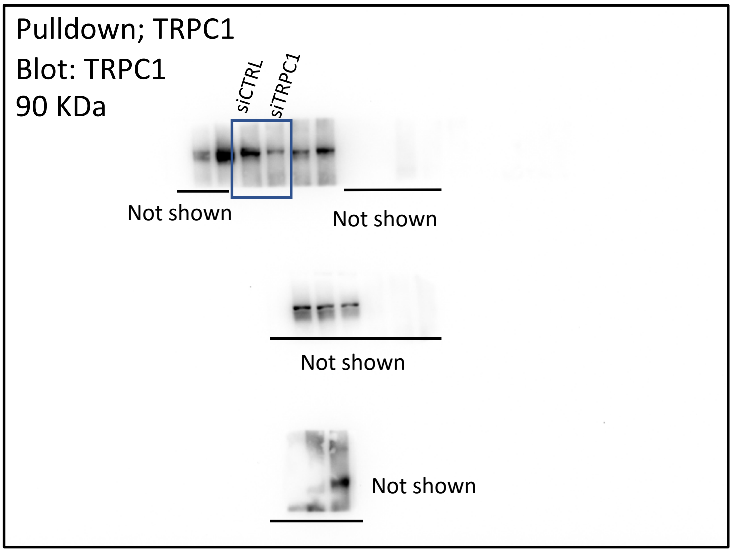

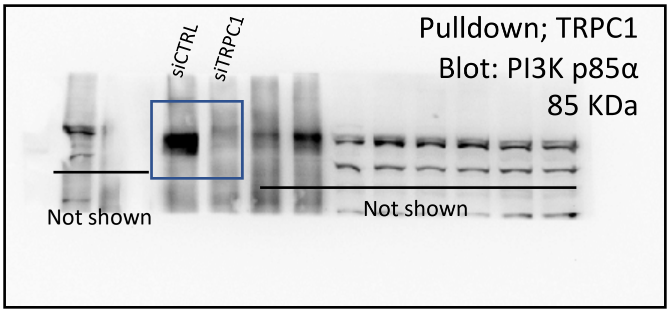

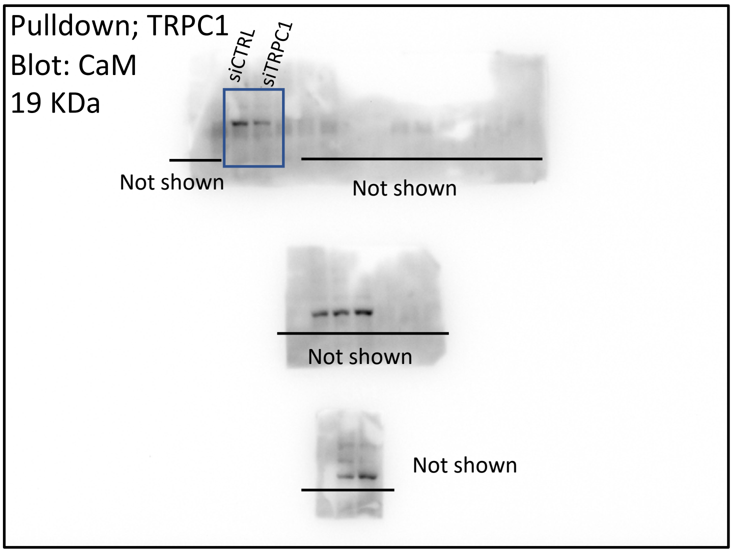

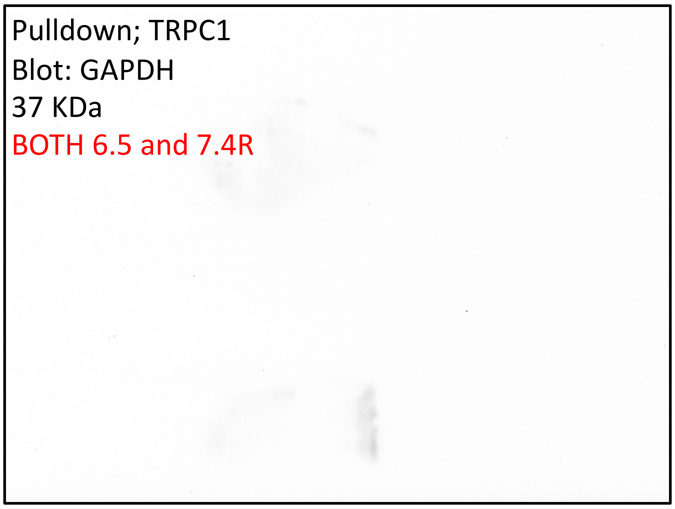

Figure 7C pH 7.4R

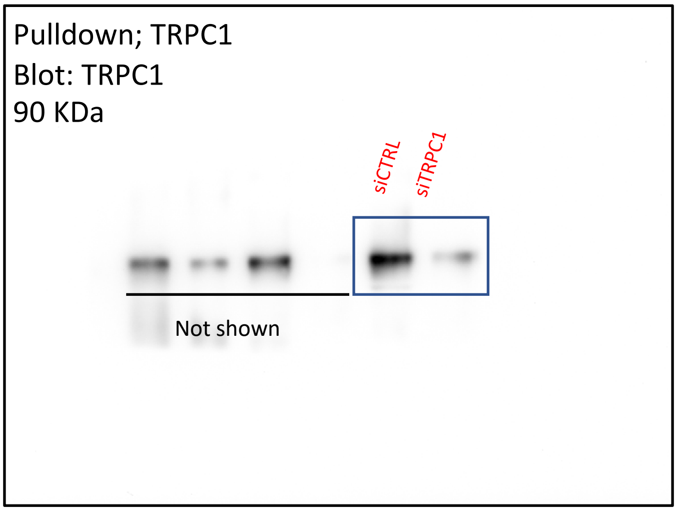

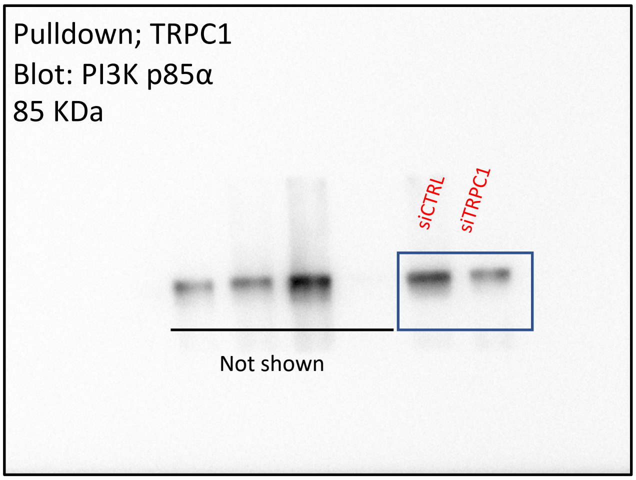

Figure 7C pH 7.4R

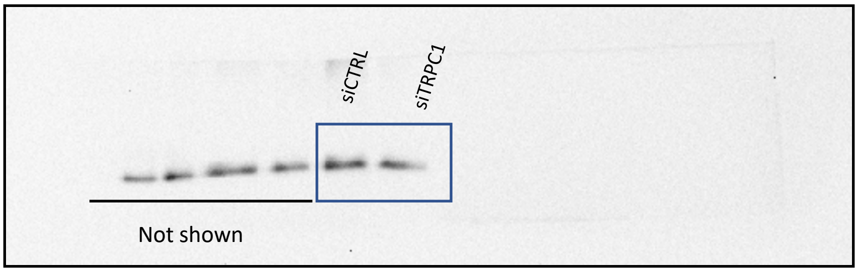

Figure 8

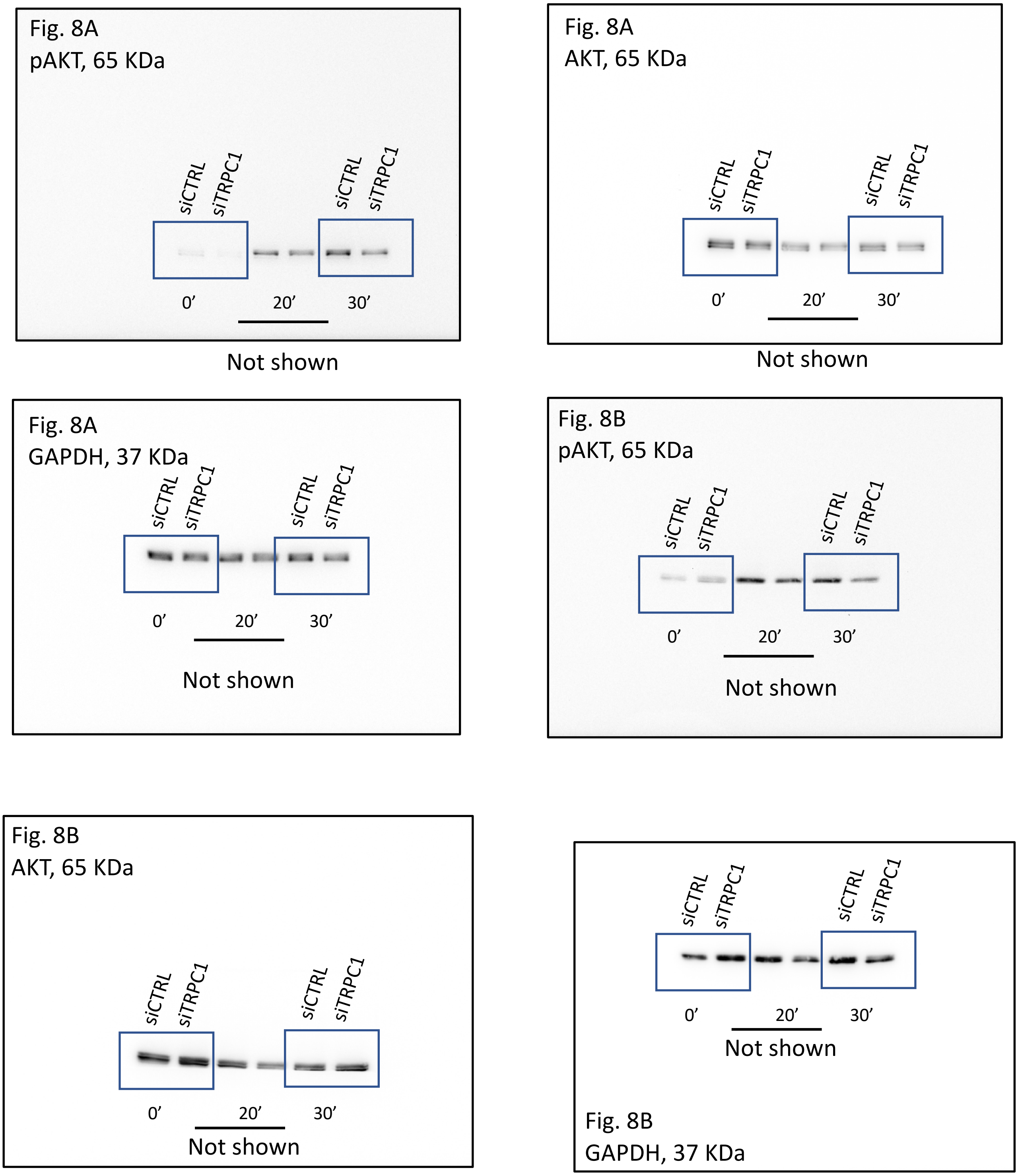

Figure 8

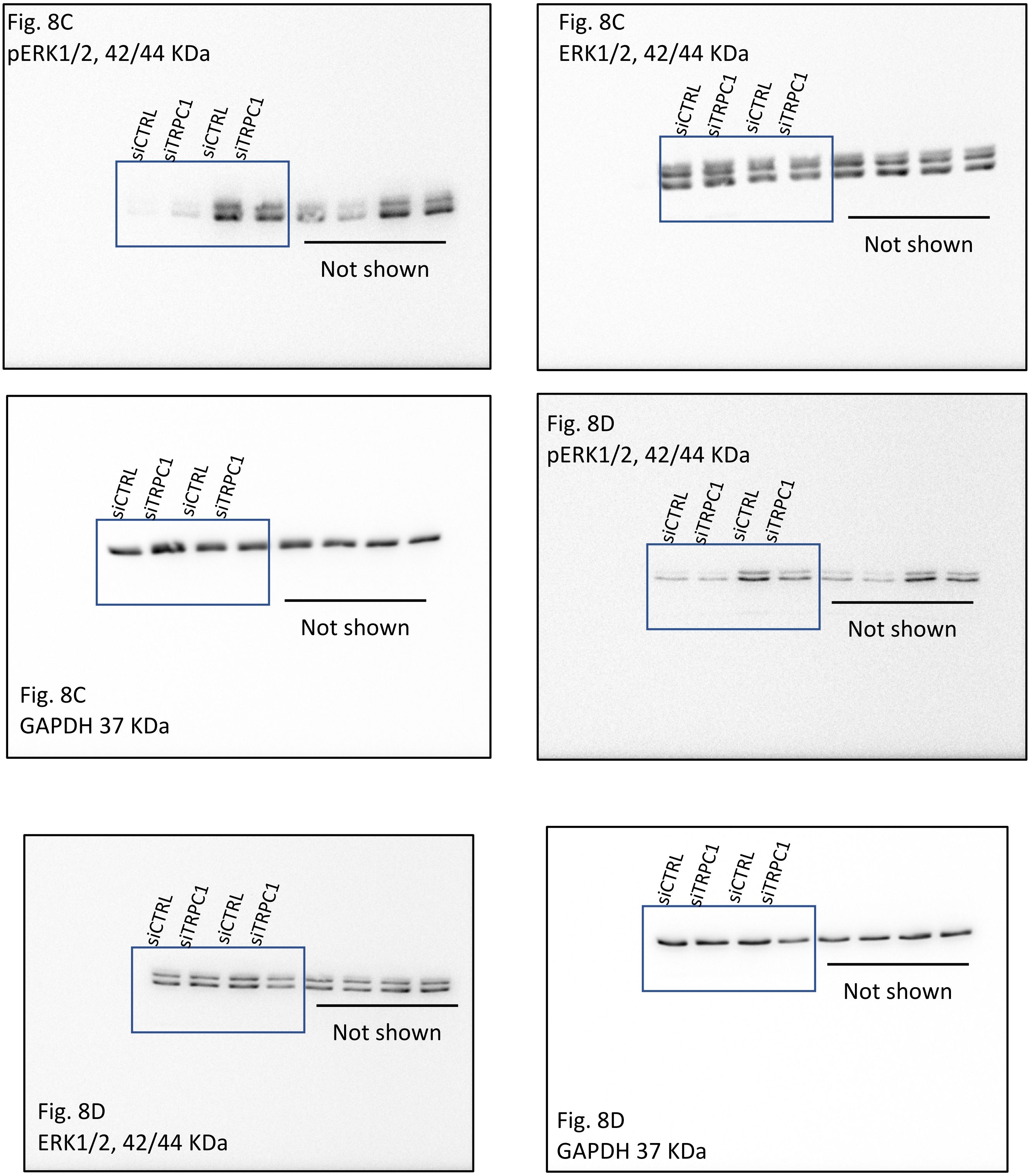
Fig. 8C
pERK1/2, 42/44 KDa
siCTRL
siTRPC1
siCTRL
siTRPC1
Not shown
Fig. 8C
ERK1/2, 42/44 KDa
siCTRL
siTRPC1
siCTRL
siTRPC1
Not shown
siCTRL
siTRPC1
siCTRL
siTRPC1
Not shown
Fig. 8C
GAPDH 37 KDa
Fig. 8D
pERK1/2, 42/44 KDa
siCTRL
siTRPC1
siCTRL
siTRPC1
Not shown
siCTRL
siTRPC1
siCTRL
siTRPC1
Not shown
Fig. 8D
ERK1/2, 42/44 KDa
siCTRL
siTRPC1
siCTRL
siTRPC1
Not shown
Fig. 8D
GAPDH 37 KDa

Supplemental Figure S4

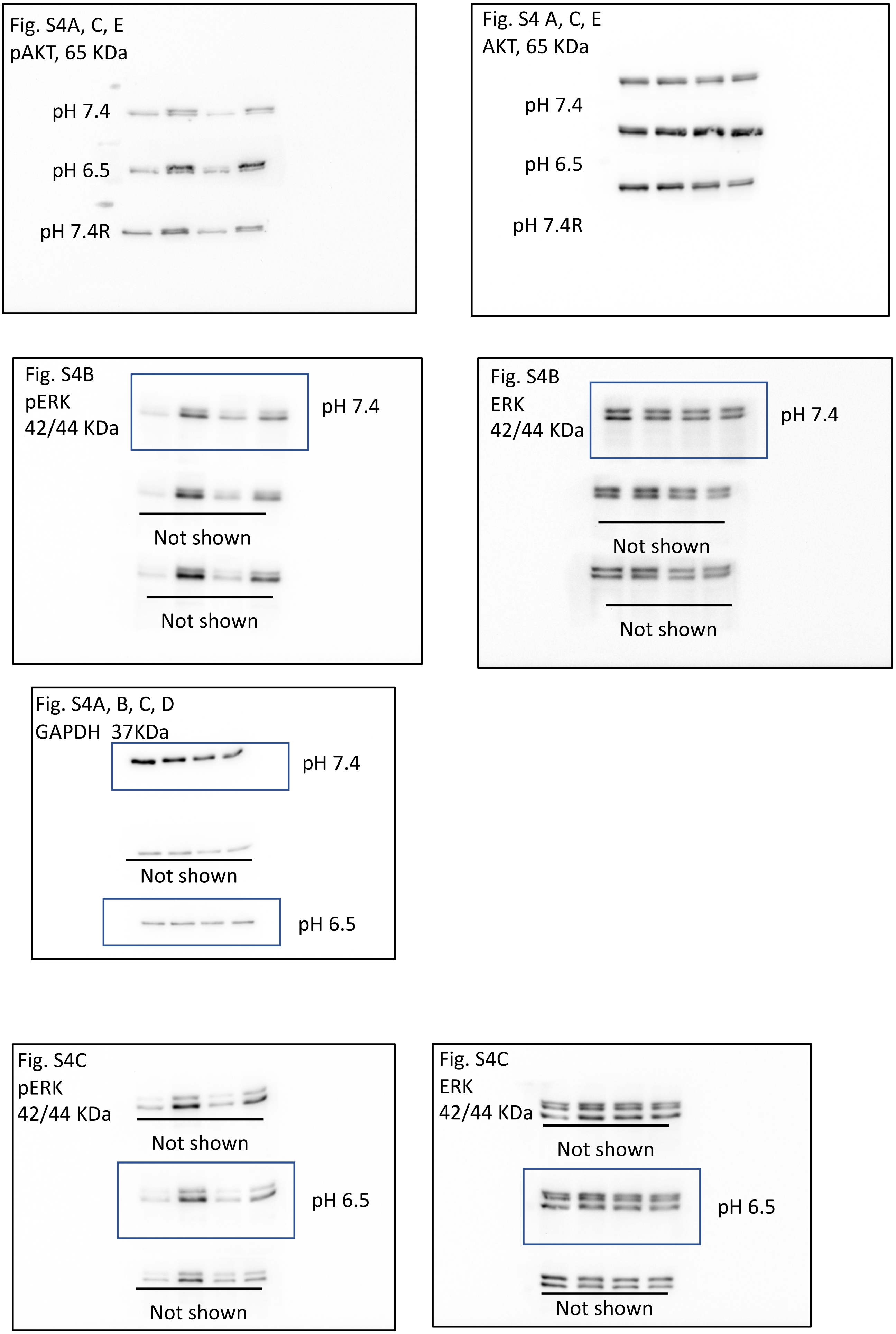

Supplemental Figure S4

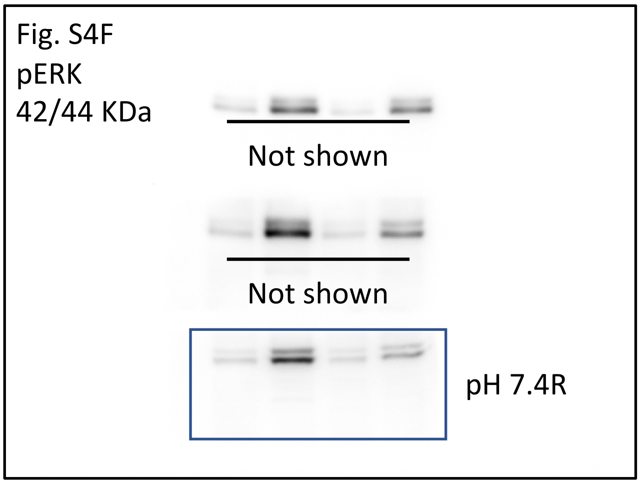

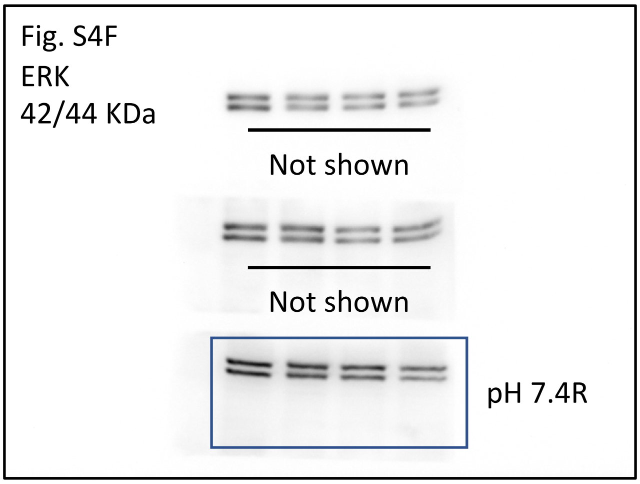

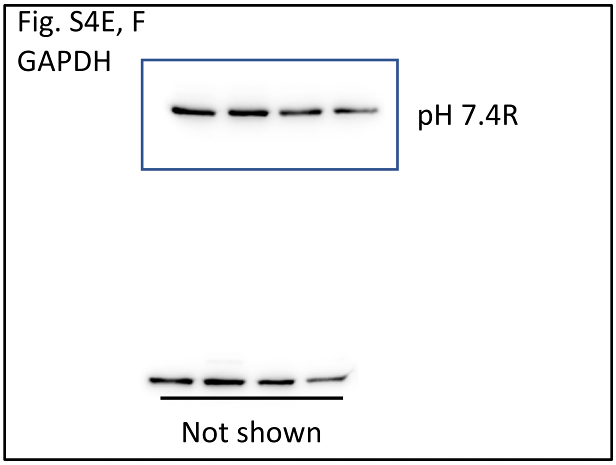

Supplement: Supplementary file 1 [file cancers-14-04946-s001.zip › Supplementary File S1.WB.pdf]
